# Genome mining identifies a diversity of natural product biosynthetic capacity in human respiratory *Corynebacterium* strains

**DOI:** 10.1128/msphere.00258-25

**Published:** 2025-05-21

**Authors:** Ashley L. Cunningham, Ilya Y. Zhbannikov, Rachel Myers, Tommy H. Tran, Wei Gao, Katherine P. Lemon, Jhoanna N. Aquino, Jillian H. Hurst, Joon Won Yoon, Patrick C. Seed, Matthew S. Kelly

**Affiliations:** 1 Department of Pediatrics, Northwestern University 309510, Chicago, Illinois, USA; 2 Stanley Manne Children’s Research Institute, Ann and Robert H. Lurie Children’s Hospital, Chicago, Illinois, USA; 3 Department of Medicine Clinical Research Unit, Duke University 3065, Durham, North Carolina, USA; 4 Alkek Center for Metagenomics and Microbiome Research, Department of Molecular Virology and Microbiology, Baylor College of Medicine 3989, Houston, Texas, USA; 5 The Forsyth Institute (Microbiology) 1816 , Cambridge, Massachusetts, USA; 6 Division of Infectious Diseases, Texas Children’s Hospital, Department of Pediatrics, Baylor College of Medicine 3989, Houston, Texas, USA; 7 Division of Pediatric Infectious Diseases, Duke University 3065, Durham, North Carolina, USA; 8 Department of Molecular Genetics and Microbiology, Duke University 3065, Durham, North Carolina, USA; The University of Iowa, Iowa City, Iowa, USA

**Keywords:** upper respiratory tract, bacteriocins, whole-genome sequencing, human microbiota, microbiome, biosynthetic gene clusters, secondary metabolites

## Abstract

*Corynebacterium* species, integral to the healthy human upper respiratory tract (URT) microbiota, remain underexplored in microbial genomics for their potential to promote respiratory health and exclude pathobionts. This genomic study investigated the diversity and capacity for natural product synthesis within these species, as indicated by their biosynthetic gene clusters (BGCs). We aimed to map and quantify the BGC diversity in a contemporary collection of *Corynebacterium* strains, representative of their prevalence in the respiratory microbiota, and to elucidate intra- and interspecies variation in BGC content. The outcomes of this research could reveal key factors in maintaining the ecological balance of the upper respiratory tract and identify novel antimicrobial agents targeting respiratory pathobionts. Employing an *in silico* approach, we analyzed the biosynthetic potential of respiratory strains of non-diphtheriae *Corynebacterium* species and their reference genomes through genome sequencing and antiSMASH6 analysis. Among 161 genomes, we identified 672 BGCs, 495 of which were unique, including polyketide synthase, non-ribosomal peptide synthetase, ribosomally synthesized and post-translationally modified peptide, and siderophore families. To understand how this biosynthetic capacity compared to other respiratory bacteria, we then downloaded genomes from eight species that are associated with the URT and conducted BGC searches. We found that despite their compact genomes, *Corynebacterium* species possess a multitude of predicted BGCs, exceeding the diversity of natural product BGCs identified in multiple other respiratory bacteria. This research lays the foundation for future functional genomics studies on the role of *Corynebacterium* species in the respiratory microbiome and the discovery of novel therapeutics derived from this bacterial genus.

**IMPORTANCE:**

Bacterial secondary metabolites, produced by enzymes encoded by biosynthetic gene clusters, are ecologically important for bacterial communication and competition in nutrient-scarce environments and are a historically rich source of antibiotics and other medications. Human-associated *Corynebacterium* species, abundant in the healthy upper respiratory tract, are understudied despite evidence of their roles in promoting human health and preventing pathobiont colonization. Through genome mining of a large collection of *Corynebacterium* strains isolated from the human respiratory tract and publicly available genomes of other respiratory bacteria, our study suggests that *Corynebacterium* species have a high biosynthetic capacity and are predicted to harbor a wide range of biosynthetic gene cluster families. These findings substantially expand current knowledge regarding the production of secondary metabolites by human-associated *Corynebacterium* species. Our study also lays the foundations for understanding how *Corynebacterium* species interact in the healthy human upper respiratory tract and the potential for discovering novel biotherapeutics.

## INTRODUCTION

*Corynebacterium* species commonly colonize the human upper respiratory tract (URT). Few of the approximately 170 *Corynebacterium* species validly published to date are known to be pathogenic with the notable exceptions of *Corynebacterium diphtheriae* and select species that infrequently produce invasive infection in highly immunocompromised patients (1–4). Previous studies have demonstrated an inverse relationship between the relative abundance of *Corynebacterium* species in the human URT and the presence of opportunistic pathogens (pathobionts) such as *Streptococcus pneumoniae* and *Staphylococcus aureus,* suggesting a protective role of *Corynebacterium* species in the respiratory environment (5–8). The inverse association with respiratory pathobionts, low pathogenicity, and ubiquity of non-diphtheriae *Corynebacterium* species in the URT underscore their importance in respiratory health, though the specific mechanisms by which *Corynebacterium* species interact with other microbial species in the URT remain largely unknown.

The complex ecosystem of the human URT carries a diverse array of bacteria and other microbes that form the respiratory microbiota (9, 10). These microbial communities are crucial for maintaining host health, partly by resisting pathobiont colonization (11–13). A key mechanism by which bacteria in the URT contribute to host health is through the production of secondary metabolites—small molecules with diverse structures and functions (14). Secondary metabolites, often produced by enzymes encoded by biosynthetic gene clusters (BGCs), are important for microbial communication and survival (15, 16). Common URT microbes, such as *Corynebacterium propinquum* and *Staphylococcus lugdunensis*, produce secondary metabolites that inhibit the growth of other bacteria inhabiting the same environment (17, 18). However, despite these prior studies, the role of secondary metabolites in microbe-microbe interactions in the URT is less well studied than in other human-associated environments, such as the gut (19). Historically, secondary metabolites produced by microbes, including actinomycetes bacteria, have been a rich source of drug discovery, contributing to the development of treatments for many human diseases (20). Over the past four decades, more than half of the antibacterial agents approved were developed from microbial natural products or their derivatives (21–23). Two BGC classes in particular have been rich sources of many medicinally important natural products: polyketide synthase (PKS) and non-ribosomal peptide synthetase (NRPS). Polyketides have a wide range of biological functions and are used in various therapeutics, including antibiotics (e.g., erythromycin A and tetracycline), chemotherapeutics (e.g., daunorubicin and doxorubicin), immunosuppressives (e.g., rapamycin), and hypercholesterolemia medications (e.g., lovastatin), and are produced by three subgroups (types I–III) of PKS (24–29). NRPSs are large, multimodular enzymes that produce structurally and functionally diverse non-ribosomal peptides (30). Important products of NRPS enzymes include antibiotics (e.g., beta-lactams, daptomycin, and vancomycin), chemotherapeutics (e.g., bleomycin), immunosuppressives (e.g., cyclosporine A), and antifungals (e.g., echinocandins) (31, 32).

Natural products from other BGC families, such as siderophores and ribosomally synthesized and post-translationally modified peptides (RiPPs), play critical roles in microbial competition and fitness. In the URT, bacteria face a scarcity of iron, which is essential for survival. Siderophores, molecules that bind and transport iron, facilitate iron scavenging and aid in competitive metal acquisition (33–35). Siderophores are synthesized through NRPS-dependent or NRPS-independent siderophore (NIS) synthetase pathways (36). Illustrating the role of siderophores in URT ecological competition, nasal strains of *C. propinquum* produce the siderophore dehydroxynocardamine that inhibits coagulase-negative *Staphylococcus* species in the nasal cavity (17).

RiPPs are small peptides known for their extensive structural variety and biological activity, which arise from a range of post-translational modifications (37). Bacteriocins, a type of RiPP with potent antimicrobial properties, often target the same or closely related species to the producing microbe and are currently being studied for their potential use as alternatives to conventional antibiotics (38, 39).

In this study, we addressed the extent to which genomes of respiratory *Corynebacterium* strains harbor a diverse array of BGCs and, consequently, the capacity to produce a range of small bioactive molecules. We employed *in silico* genome mining to screen 148 *Corynebacterium* strains isolated from human respiratory samples collected on two continents and their reference genomes for natural product BGCs. We identified 495 unique BGCs of 672 total, including BGCs belonging to the PKS, NRPS, siderophore, and RiPP gene families. To understand if the diverse BGC capacity is unique to *Corynebacterium* among URT bacteria, we performed genome mining on publicly available genomes from other URT commensal bacteria and pathobionts. We found that the biosynthetic capacity of non-diphtheriae *Corynebacterium* strains was more diversified compared to multiple other common species, but not all, that associate with the URT. These findings suggest that *Corynebacterium* species that are common to the URT harbor BGCs that may yield inhibitory molecules against important human pathobionts, influencing the URT environment. Furthermore, these *Corynebacterium* species may be a rich source of natural products with biotherapeutic potential for preventing respiratory infections.

## RESULTS

### Description of non-diphtheriae *Corynebacterium* strains

The analyzed isolate collection contained 148 strains representing 13 non-diphtheriae *Corynebacterium* species. The sources of the strains were as follows: (i) nasopharyngeal samples collected from infants and mothers in a study that was conducted in Botswana (*n* = 60); (ii) patient respiratory samples received by the Duke University Health System Clinical Microbiology Laboratory (*n* = 12); and (iii) nostril swabs donated by participants at scientific outreach events in Massachusetts (*n* = 76). Species were assigned based on average nucleotide identity (ANI) calculations compared to the reference genomes of *Corynebacterium* species using the standards proposed by Chun et al. (7, 40–43). Ten out of the 13 reference genomes for the species representing the respiratory *Corynebacterium* strains come from non-human or non-respiratory isolation sources (Table S1). To assess how these *Corynebacterium* species, which are associated with the human URT but also colonize other niches, may differ based on isolation source, the reference genome for each assigned *Corynebacterium* species was downloaded and included in subsequent analyses. The average genome size was 2,444,000 base pairs (bp; range: 2,155,492–2,977,329 bp). The *Corynebacterium* species *pseudodiphtheriticum* (*n* = 39), *accolens* (*n* = 31), *propinquum* (*n* = 15), and *marquesiae* (*n* = 10), plus isolates matching the genome reported as *Corynebacterium kefirresidentii* (*n* = 9), accounted for the majority of strains (Table 1).

**TABLE 1:** Non-diphtheriae *Corynebacterium* species included in this study^*a*^

Species	Number of genomes
*Corynebacterium pseudodiphtheriticum*	39
*Corynebacterium accolens*	31
*C. propinquum*	15
*Corynebacterium marquesiae*	10
*C. kefirresidentii*	9
*Corynebacterium striatum*	5
*Corynebacterium yonathiae*	3
*Corynebacterium appendicis*	2
*Corynebacterium bovis*	2
*Corynebacterium coyleae*	2
*Corynebacterium freneyi*	2
*Corynebacterium mastitidis*	2
*Corynebacterium tuberculostearicum*	2
Unknown	37
Total	161

^*a*^ The *Corynebacterium* species and number of genomes per species included in these analyses are shown. The number of genomes includes all the respiratory *Corynebacterium* strains and the reference genome for each species*.* Each genome comes from a distinct strain.

### Overview of biosynthetic gene clusters in non-diphtheriae *Corynebacterium* strains

**Fig 1:**
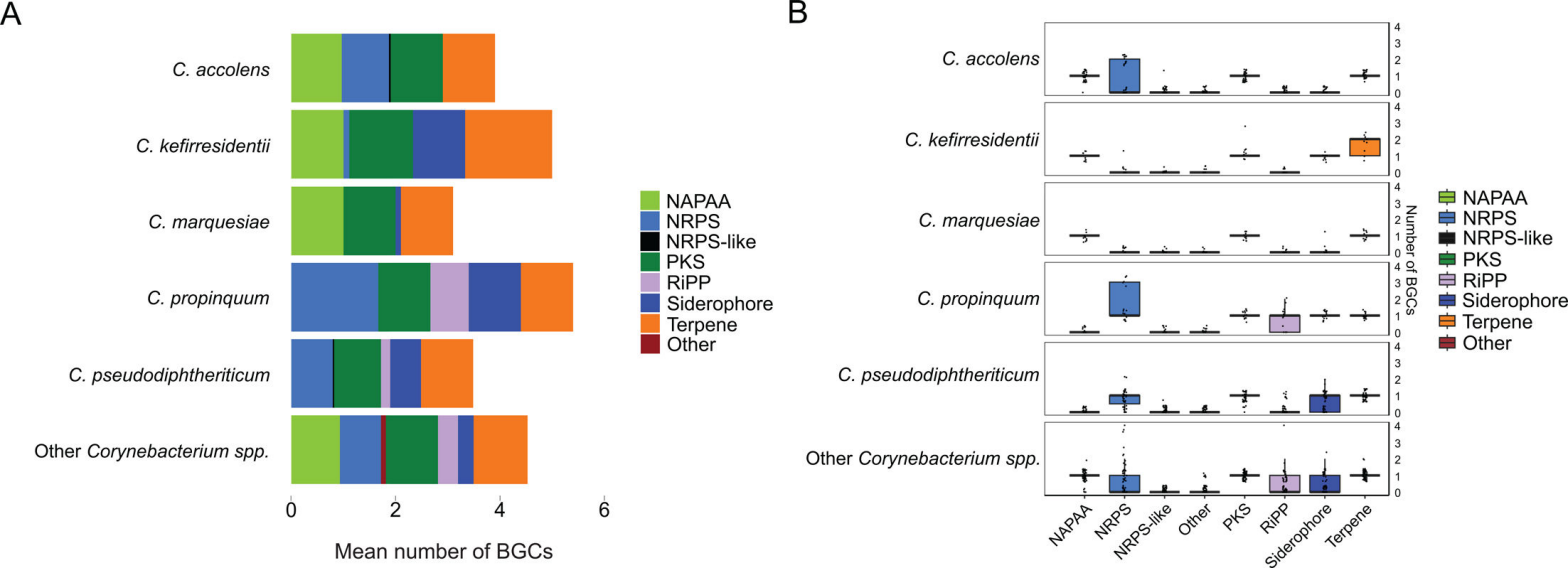
Overview of the biosynthetic capacity and strain-level variation of common BGC families identified in non-diphtheriae *Corynebacterium* genomes. (**A**) Bar plots depicting the mean number of BGCs for the most common BGC families across the genomes of the respiratory strain collection and 13 reference genomes. Only species represented by nine or more genomes are depicted separately. (**B**) Box plots depicting the strain-level variation in the biosynthetic capacity of the most common species for predicted BGC families. Each dot represents a single strain. The bottom and top edges of the boxes represent the 25th and 75th percentiles, respectively.

Using antibiotics and secondary metabolite analysis shell version 6.0 (antiSMASH6), we conducted BGC searches, identifying 672 BGCs, 495 of which were unique, across the 148 respiratory *Corynebacterium* genomes and 13 reference genomes (161 genomes total), with a median (interquartile range) of 4 (3–5) BGCs per genome (Table S2) (44). The most common gene families were terpene (*n* = 169), PKS (*n* = 159), non-alpha poly-amino acid (NAPAA; *n* = 102), and NRPS (*n* = 130). BGC distribution varied among *Corynebacterium* species (Fig. 1A). Terpene, PKS, NAPAA, and NRPS gene clusters were found across *Corynebacterium* species, except that NAPAA gene clusters were absent from the closely related species *C. propinquum* and *Corynebacterium pseudodiphtheriticum*, and NRPS gene clusters were absent from *Corynebacterium marquesiae*. Putative siderophore BGCs (*n* = 65) were mainly found in *C. kefirresidentii*, *C. propinquum*, and *C. pseudodiphtheriticum*. We identified 40 putative RiPP gene clusters in 33 genomes*,* which included class IId bacteriocins (*n* = 15), linardins (*n* = 10), lanthipeptides (*n* = 6), DUF692-associated bacteriocins (*n* = 2), linear azol(in)e-containing peptides (LAPs; *n* = 2), thiopeptides (*n* = 2), lasso peptides (*n* = 2), and RiPP recognition element (RRE) domain-containing clusters (*n* = 1). We then looked at the variation in the biosynthetic capacity of the most common species (represented by greater than equal to nine genomes for a given species), when broken down by source (Botswana, US, or National Center for Biotechnology Information [NCBI] reference genome). We found that the number and identity of BGC clusters harbored by given species varied by the source of the genome (Fig. S1). Overall, we observed that all the highly represented species were predicted to harbor at least two BGC classes among their genomes, suggesting that these *Corynebacterium* species have the capacity to produce natural products, regardless of their source.

To understand strain-level variation in biosynthetic capacity of natural products, we examined in detail the predicted BGC classes in the *Corynebacterium* species most represented among the 161 genomes (greater than equal to nine genomes for a given species; Fig. 1B). The number of BGCs per genome for each BGC class was similar among *C. marquesiae*, *C. kefirresidentii*, and *Corynebacterium accolens*, aside from predicted siderophore clusters in *C. kefirresidentii* and NRPS clusters in *C. accolens*. Conversely, *C. pseudodiphtheriticum* and *C. propinquum* strains demonstrated more variation in biosynthetic capacity. The number of NRPS and RiPP BGCs varied per strain among *C. propinquum*, while the number of NRPS and siderophore BGCs per strain varied across *C. pseudodiphtheriticum*. Overall, by using genome mining to identify putative BGCs harbored within the genomes of 148 respiratory strains and 13 reference genomes, we found that respiratory *Corynebacterium* strains harbor a variety of BGC classes, with some variation between strains of the same species and between isolation sources.

### Overview of biosynthetic gene clusters in respiratory strains of other bacterial species

To assess how the biosynthetic capacity of the respiratory non-diphtheriae *Corynebacterium* strains compares to other bacterial species that are also associated with the human URT environment, we assembled and conducted BGC searches on a collection of publicly available genomes from respiratory strains of a set of bacteria that associate with the human URT (Table S3). Up to 40 genomes, depending on the number of genomes available for a given species, plus the reference genomes for each species were used in the analysis (*n* = 269). The species used for the BGC search included the pathogen *C. diphtheriae*, gram-negative pathobionts (*Moraxella catarrhalis* and *Haemophilus influenzae*), gram-positive pathobionts (*S. pneumoniae* and *S. aureus*), and gram-positive commensals (*Dolosigranulum pigrum*, *Streptococcus mitis*, and *Staphylococcus epidermidis*; Table 2).

**TABLE 2:** Other respiratory bacterial strains included in this study^*a*^

Species	Number of genomes
*H. influenzae*	41
*M. catarrhalis*	41
*S. aureus*	41
*S. pneumoniae*	41
*S. epidermidis*	38
*C. diphtheriae*	33
*D. pigrum*	25
*S. mitis*	9
Total	269

^*a*^ The species of respiratory strains and number of genomes per species that were downloaded and included in this study. Up to 40 genomes from each species, plus the reference genome, make up the total number of genomes. Each genome comes from a distinct strain.

**Fig 2:**
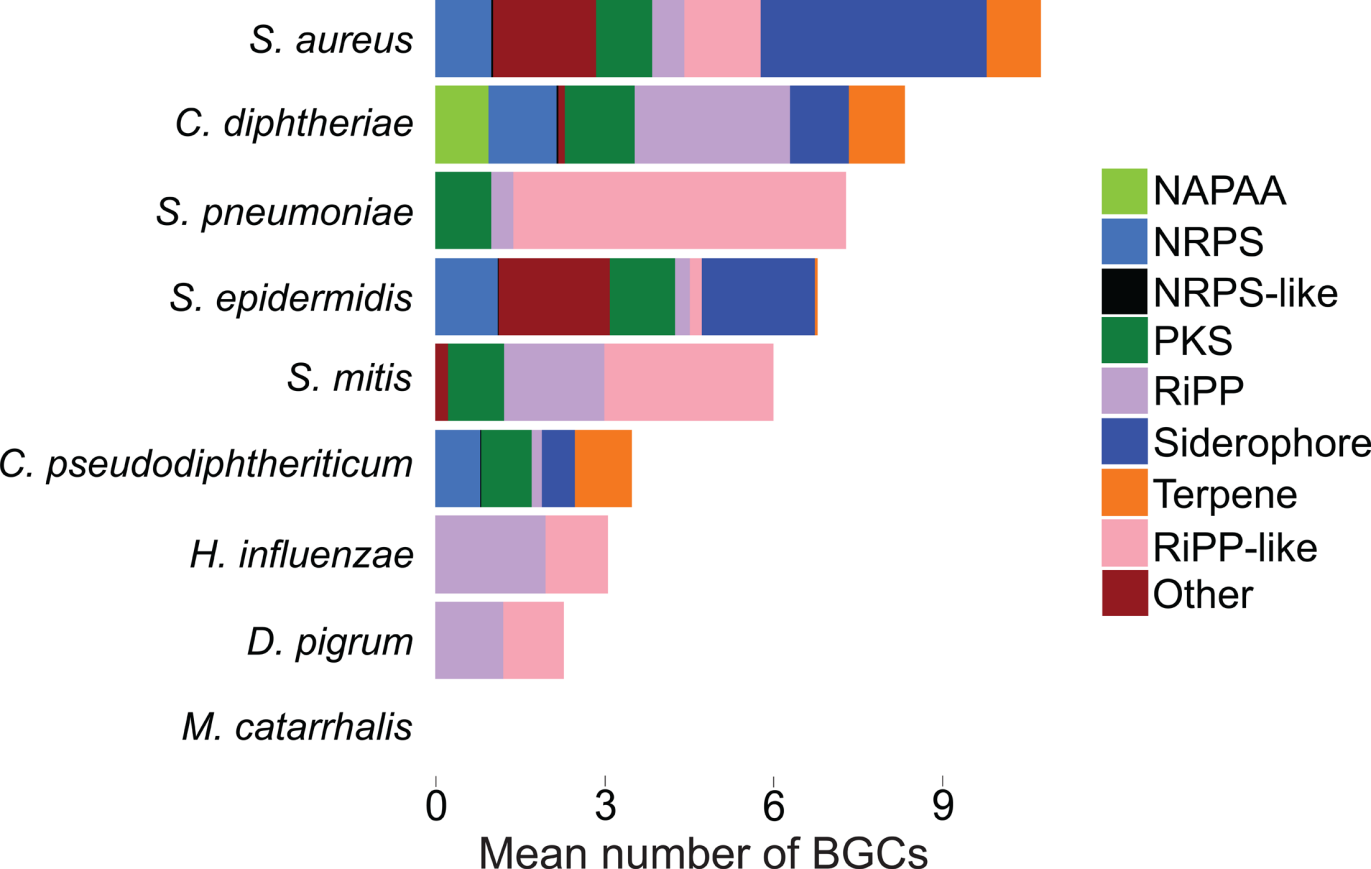
Overview of the biosynthetic capacity identified in respiratory strains of other bacterial species. Bar plots depicting the mean number of BGCs for the most common BGC classes across the genomes of respiratory strains of URT-associated bacterial species and their reference genomes. The species *C. pseudodiphteriticum*, which was highly represented among the non-diphtheriae *Corynebacterium* species genomes, is included for comparison.

Genome mining revealed a spread in biosynthetic capacity among these bacterial species (Fig. 2). A total of 1,510 BGCs were identified among the 269 genomes, with a median (interquartile range) of 4.5 (1–8) per genome. Two of the 25 *D. pigrum* genomes and all 41 *M*. *catarrhalis* genomes screened were predicted to harbor no BGCs. RiPP-like (*n* = 406), siderophore (*n* = 274), and RiPP (*n* = 266) BGCs were the most abundant BGC types (Table S4). Other common BGC families included cyclic lactone autoinducer (*n* = 149), PKS (*n* = 135), and NRPS (*n* = 123). Unlike in the non-diphtheriae *Corynebacterium* genomes, terpene (*n* = 75) and NAPAA (*n* = 31) clusters were relatively less common. The specific class and number of BGC classes varied widely between species and genus. The species with the greatest predicted diversity in biosynthetic capacity were *S. aureus*, *S. epidermidis*, and *C. diphtheriae*, which were each predicted to harbor at least eight BGC families in their genomes. The mean number of BGCs per species was higher for most of the analyzed species than that of *C. pseudodiphtheriticum*, which was included as a representative non-diphtheriae *Corynebacterium* species. *C. pseudodiphtheriticum* was chosen as a representative species because it was represented by the highest number of genomes (*n* = 39) among the non-diphtheriae *Corynebacterium* species and was predicted to harbor a diverse spread of BGC types. Only *H. influenzae*, *D. pigrum*, and *M. catarrhalis* had a lower mean number of BGCs per species compared to *C. pseudodiphtheriticum*. However, the diversity of BGC classes identified among *C. pseudodiphtheriticum* was greater than the diversity from five (*S. pneumoniae*, *S. mitis*, *H. influenzae*, *D. pigrum*, and *M. catarrhalis*) of the eight species analyzed, as measured by distinct types of predicted BGC families. Overall, genome mining of genomes from a diverse set of bacteria that colonize the URT revealed a spread in biosynthetic capacity, with multiple species predicted to harbor a smaller diversity in BGC families compared to the non-diphtheriae respiratory *Corynebacterium* genomes from our contemporary collection.

### Analysis of important BGC classes in respiratory non-diphtheriae *Corynebacterium* strains

#### Identification of polyketide synthase gene clusters

**Fig 3:**
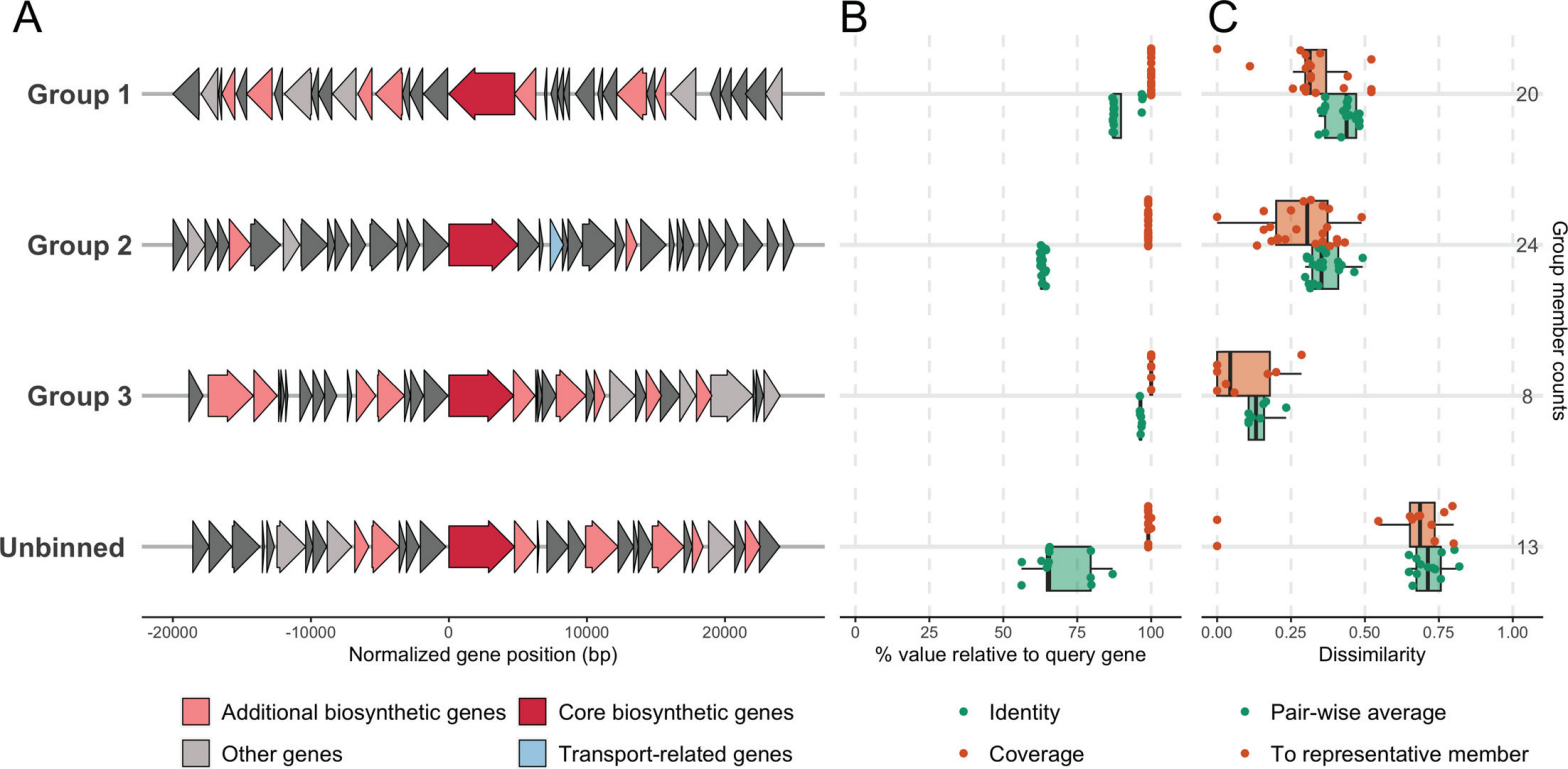
Genetic variation in T1PKS BGCs from non-diphtheriae *Corynebacterium* genomes. (**A**) Representative member genetic architecture of each identified group from predicted T1PKS clusters in respiratory *Corynebacterium* strains and reference genomes. “Unbinned” refers to all gene clusters that the GeneGrouper algorithm did not bin into any discrete group. The core gene annotation and predicted protein product for each representative member cluster are as follows: groups 1, 3, and unbinned: *ppsA*, phthiocerol synthesis PKS type I PpsA. Group 2: *pks13*, PKS Pks13. (**B**) Coverage (orange) and amino acid identity (green) of each member of the gene cluster groups in comparison to the initial query genes (the seed genes). Coverage refers to the proportion of the query gene sequences that each member of the group covers. Amino acid identity represents the similarity of the amino acid sequence between the query genes and each group member, expressed as a percentage. The seed genes were taken from BGCs not located on contig edges and were core BGC genes. (**C**) Plot of two measures of group member gene content dissimilarity: (i) Jaccard dissimilarity (dissimilarity of each member relative to the group representative) of each group’s members. A low Jaccard dissimilarity indicates high similarity, while a high Jaccard dissimilarity indicates low similarity between the member and the group representative (orange). (ii) The average pairwise dissimilarity of each member to all others within the same group (green). This provides an understanding of the overall diversity or variation within the gene group. A low average pairwise dissimilarity suggests that the members are relatively similar, while a high average pairwise dissimilarity suggests that the members are more diverse.

The overwhelming majority (98%) of the 159 identified PKS gene clusters were T1PKS. One unspeciated strain carried a T3PKS, and two genomes from *C. kefirresidentii* encoded a *trans*-AT PKS. GeneGrouper was applied to understand the variation among the T1PKS clusters (45). This tool categorizes large sets of BGCs into smaller, discrete groups. To ensure the grouping of only distinct and complete T1PKS cluster sequences, the T1PKS BGCs were narrowed down to 66 unique, non-contig edge (not fragmented across multiple contigs) T1PKS gene clusters. Using these T1PKS gene clusters as seed genes, GeneGrouper binned the T1PKS clusters into three discrete groups, as well as an unbinned group consisting of clusters not genetically similar to clusters in groups 1 through 3 (Fig. 3A). The genetic similarity of all clusters within each group (1, 2, 3, or unbinned) was visualized using Easyfig (Fig. S2A through D) (46). While the clusters in groups 1, 2, and 3 showed similarity between each member of the group, the clusters in the unbinned group did not exhibit high similarity.

GeneGrouper also provides information about the coverage and amino acid identity of each member of a gene cluster group compared to the translated query genes (Fig. 3B), as well as the dissimilarity in full gene cluster content between each member in a given group to each other and to a representative member of the group (Fig. 3C). Generally, there was high coverage between the query genes and the genes of each member within the three discrete groups, with less similarity between amino acid identities. Nonetheless, the amino acid identity was greater than 75% for two of the three binned groups, suggesting that among the grouped T1PKS clusters, both gene coverage and protein products of each group member may be similar within each group to the query gene and its translated sequence (Fig. 3B).

#### Identification of non-ribosomal peptide synthetase gene clusters

**Fig 4:**
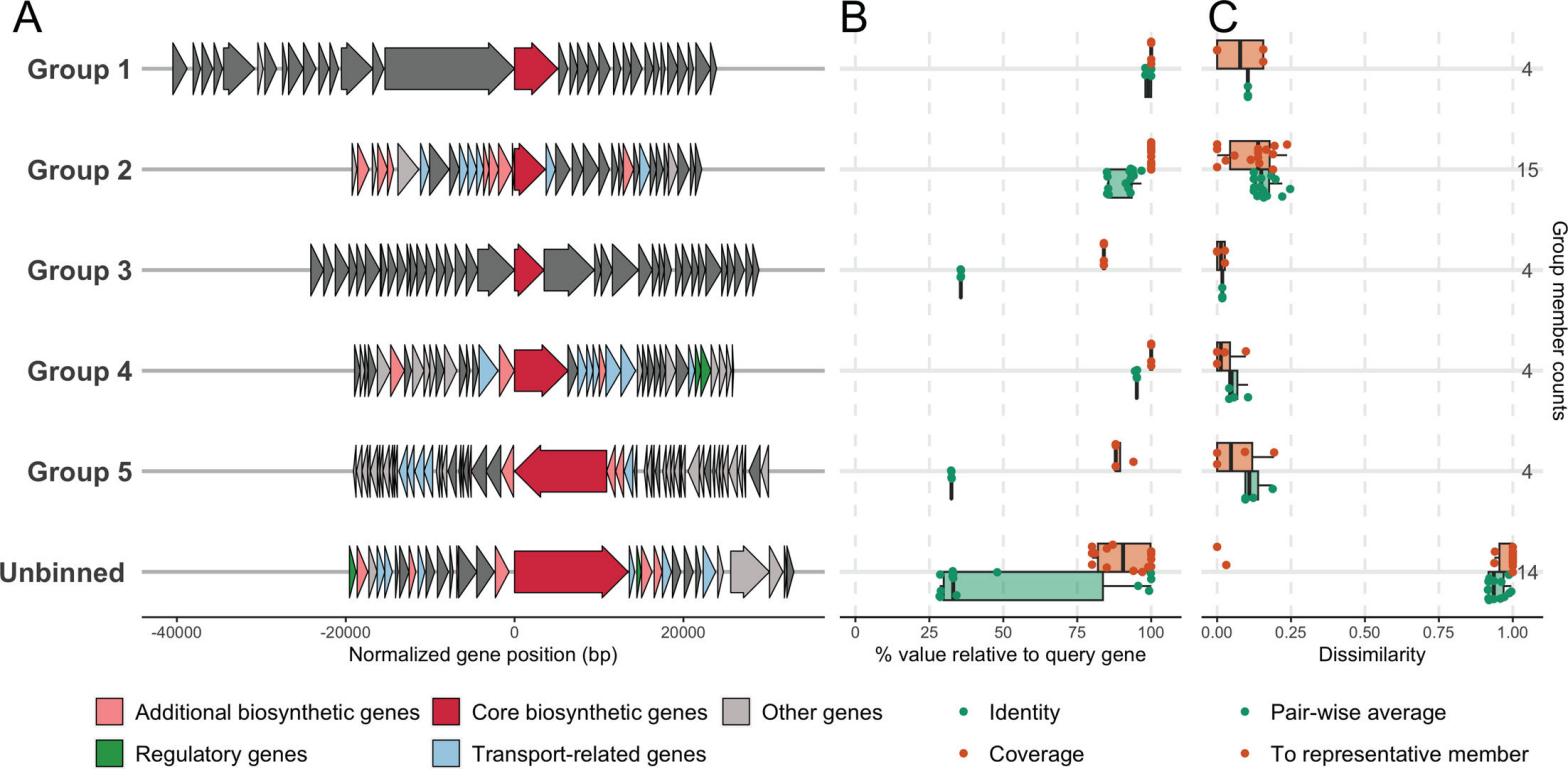
Genetic variation in NRPS BGCs from non-diphtheriae *Corynebacterium* genomes. (**A**) Representative member genetic architecture of each identified group from predicted NRPS clusters in respiratory *Corynebacterium* strains and reference genomes. The core gene annotation, where available, and predicted protein product for each representative member cluster are as follows. Group 1: condensation domain-containing protein. Group 2: *entF*, enterobactin synthase component F. Group 3: NRPS. Group 4: *lgrB*, linear gramicidin synthase subunit B. Unbinned: *lgrD_4*, linear gramicidin synthase subunit D. (**B**) Coverage (orange) and amino acid identity (green) of each member of the gene cluster groups in comparison to the initial query genes (the seed genes). (**C**) Plot of two measures of group member gene content dissimilarity: (i) Jaccard dissimilarity (dissimilarity of each member relative to the group representative) of each group’s members (orange); (ii) the average pairwise dissimilarity of each member to all other members within the same group (green).

Using antiSMASH6, we identified 130 NRPS clusters. GeneGrouper binned 45 unique, non-contig edge NRPS clusters into five discrete groups and a sixth unbinned group (Fig. 4A). The genetic similarity between the clusters within groups (1, 2, 3, 4, 5, and unbinned) was visualized with Easyfig (Fig. S3A through E). Similar to what was observed among the T1PKS cluster groups, the members of groups 1 through 5 were more similar to each other compared to the clusters in the unbinned group. Coverage in groups 1 through 5 compared to the query genes was high. In contrast to the T1PKS groups, the amino acid identity between members of the NRPS groups compared to the translated sequence of the query gene varied greatly between groups (~25% to ~100%), indicating that despite high coverage between the query gene and group members, the protein products may vary significantly in some groups (Fig. 4B). Low dissimilarity existed between the gene content of each NRPS member to its group representative, as well as low average pairwise dissimilarities, suggesting that the members of each group were similar, as measured by gene content of the entire cluster (Fig. 4C).

#### Identification of siderophore gene clusters

**Fig 5:**
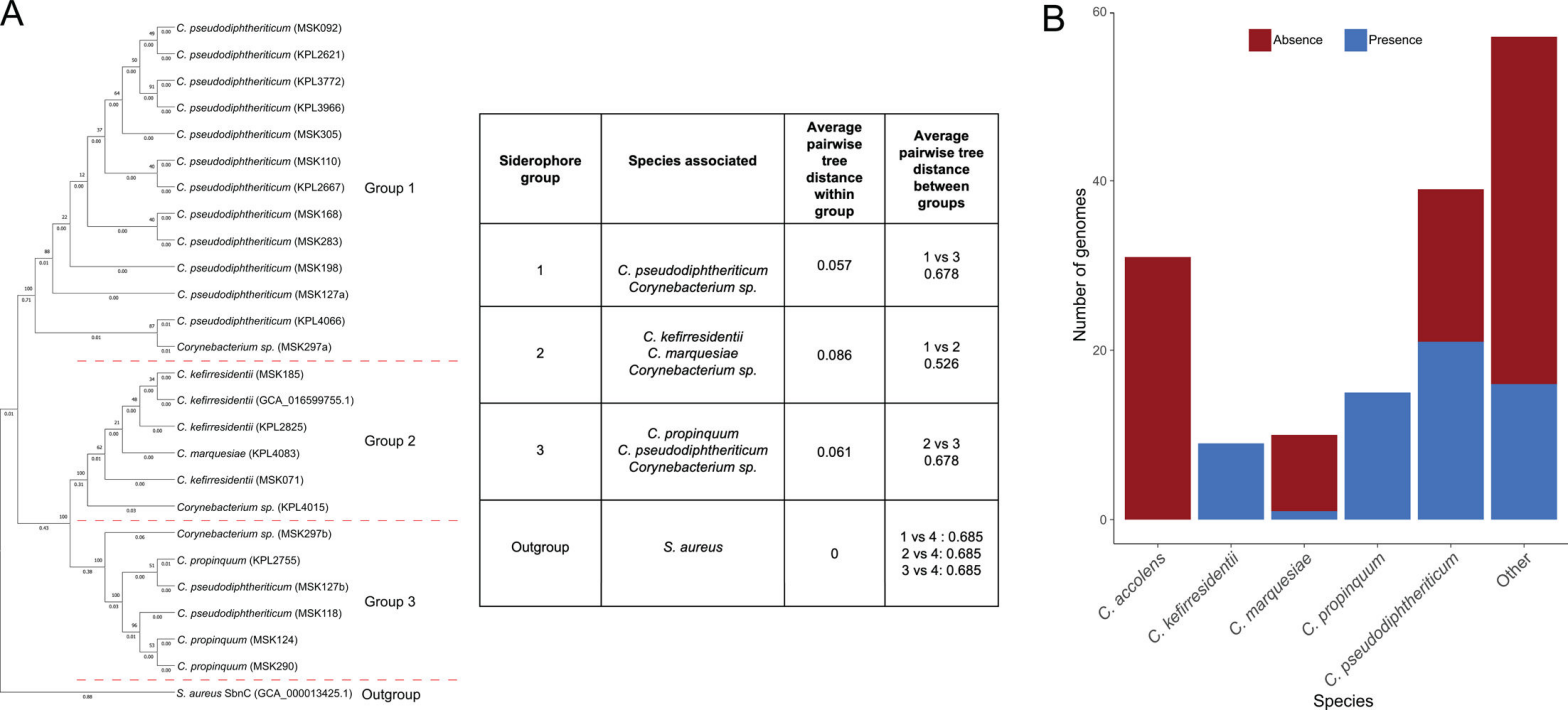
Phylogenetic relationship of predicted siderophore core biosynthetic enzymes and siderophore BGC encoding potential in non-diphtheriae *Corynebacterium* genomes. (**A**) Dendrogram depicting the phylogenetic relationship of the translated sequences of unique predicted NIS synthetase core biosynthetic genes from non-diphtheriae *Corynebacterium* genomes. The outgroup is the staphyloferrin B synthase protein (SbnC) from *S. aureus*. Dashed red lines indicate the breakpoint between the three identified groups of predicted NIS synthetase core biosynthetic enzymes based on amino acid sequence alignments. “A” and “B” designations in strain names indicate siderophore core biosynthetic enzymes stemming from distinct siderophore BGCs harbored by the same genome. The evolutionary history was inferred using the neighbor-joining method. The percentage of replicate trees in which the associated taxa clustered together in the bootstrap test (500 replicates) is shown next to the branches (48). The evolutionary distances were computed using the Poisson correction method and are in the units of the number of amino acid substitutions per site (49). The table shows the species associated with the three groups and their relative similarities. The average pairwise tree distances indicate the average similarity of predicted siderophore core biosynthetic enzyme sequences within each group and between the groups. (**B**) Bar plot indicating the potential of a given species to harbor a predicted siderophore gene cluster. Species with at least nine representative genomes are included.

Sixty-five siderophore gene clusters, defined by antiSMASH6 as encoding NIS synthetases for IucA/IucC-like siderophores, were identified across various *Corynebacterium* species. Besides *C. propinquum* (*n* = 15), strains of *C. pseudodiphtheriticum* (*n* = 21), *C. kefirresidentii* (*n* = 9), *C. marquesiae* (*n* = 1), and some unspeciated strains (*n* = 16) were found to harbor these clusters. Of these, one *C. pseudodiphteriticum* genome and one genome from an unspeciated strain were each predicted to harbor two distinct siderophore BGCs. To understand diversity in the predicted siderophore biosynthesis enzymes, we performed phylogenetic analysis and amino acid sequence alignment of the full translated sequence of the predicted core gene of each unique NIS synthetase BGC, which revealed three distinct groups (Fig. S4). The proteins in group 1, annotated by BLAST as IucA/IucC family proteins, came from *C. pseudodiphteriticum* strains and an unassigned strain that was most closely related to *C. propinquum*, based on ANI calculations (Fig. 5A) (43). The proteins in group 2 came from *C. kefirresidentii* strains, as well as a *C. marquesiae* strain and an unassigned strain most closely related to *C. accolens*, and were annotated by BLAST as GNAT family N-acetyltransferases. Group 3 proteins were annotated by BLAST as GNAT family N-acetyltransferases which came from *C. pseudodiphtheriticum* and *C. propinquum* strains. The average tree distance within these groups suggested that the core siderophore biosynthetic proteins in group 1, encoded almost entirely by *C. pseudodiphtheriticum* strains, were the most similar. In contrast, group 2 core siderophore biosynthetic enzymes, found mainly in *C. kefirresidentii*, showed the greatest diversity. The core biosynthetic enzyme for the *S. aureus* siderophore staphyloferrin B, SbnC, was included as the outgroup in the phylogenetic analysis. It was selected as the outgroup because it belongs to NIS synthetase type B, while the putative *Corynebacterium* IucA/IucC-like NIS synthetases are predicted to be either type A or type C enzymes (47).

An analysis of the presence of putative siderophore BGCs in the most common species represented among the 161 *Corynebacterium* genomes (greater than or equal to nine genomes for a given species) revealed variability in encoding potential between species. While a proportion of *C. marqeusiae* and *C. pseudodiphtheriticum* strains was predicted to harbor siderophore BGCs, all strains of *C. kefirresidentii* and *C. propinquum* contained predicted siderophore clusters within their genomes (Fig. 5B). Of the five most common species in the collection, *C. accolens* was the only species not predicted to harbor siderophore BGCs. Overall, the number of siderophore BGCs identified among the *Corynebacterium* genomes was relatively small compared to other BGC classes, including terpene, NRPS, and PKS families. However, more than one core biosynthetic enzyme for siderophore synthesis was predicted, suggesting *Corynebacterium* species could produce more than one type of siderophore (50).

#### Identification of ribosomally synthesized and post-translationally modified peptide gene clusters

**Fig 6:**
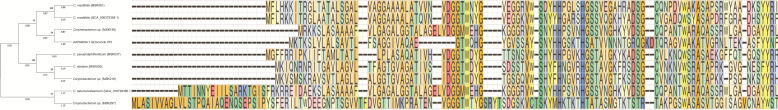
Multiple sequence alignment of class IId bacteriocins from non-diphtheriae *Corynebacterium* genomes. Dendrogram and multiple sequence alignment of the translated core biosynthetic gene sequence from all the unique predicted class IId bacteriocins identified by antiSMASH6. The core peptide sequence of class IId bacteriocins is unmodified, indicating that the mature peptide sequence of the final secondary metabolite will be the same as in the precursor peptide. The amino acid sequence for the previously characterized class IId lactococcin 972 is included for comparison. The evolutionary history was inferred using the neighbor-joining method. The percentage of replicate trees in which the associated taxa clustered together in the bootstrap test (500 replicates) is shown next to the branches (48). The evolutionary distances were computed using the Poisson correction method and are in the units of the number of amino acid substitutions per site (49). The less-saturated colors in the amino acid alignment indicate weaker conservation of amino acid residues at a given site, while more-saturated colors correspond to higher conservation.

Our analysis revealed a modest number of putative RiPP gene clusters (*n* = 40) among 33 non-diphtheriae *Corynebacterium* genomes. These clusters are predicted to encode various classes of bacteriocins, including class IId bacteriocins (*n* = 15), linardins (*n* = 10), lanthipeptides (*n* = 6), DUF692-associated bacteriocins (*n* = 2), LAPs (*n* = 2), thiopeptides (*n* = 2), lasso peptides (*n* = 2), and RRE domain-containing clusters (*n* = 1). Fig. 6 shows the alignment of core peptide amino acid sequences for all the unique predicted class IId bacteriocins. We focused on this class of RiPPs due to their abundance and the fact that they are largely unmodified, meaning the core peptide sequence remains unchanged in the mature peptide, which is the final secondary metabolite product (51). The alignment revealed that the predicted class IId bacteriocins from various *Corynebacterium* species, including *Corynebacterium tuberculostearicum*, *Corynebacterium striatum*, *C. pseudodiphtheriticum*, and *Corynebacterium mastitidis*, share homology to the previously described bacteriocin lactococcin 972 (52). Additionally, unique class IId bacteriocins were identified among the reference genomes of *C. mastitidis* (GCA_000375365.1) and *C. tuberculostearicum* (GCA_016728365.1) that were not predicted in the genomes of respiratory strains of the same species from our collection.

To assess the novelty of the putative *Corynebacterium* class IId bacteriocins, a discontiguous megablast search, excluding the *Corynebacterium* genus, was performed for each of the eight *Corynebacterium* core biosynthetic genes encoding the class IId bacteriocins depicted in Fig. 6. Of the eight genes, only two, both from *C. mastitidis*, returned hits outside of the *Corynebacterium* genus. The top hits for the core biosynthetic gene from the reference genome of *C. mastitidis* (GCA_000375365.1) mapped to strains of *Streptomyces europaeiscabiei* with 39% query coverage and 74% identity. For the core biosynthetic gene from *C. mastitidis* MSK081, the top hit mapped to the genome of an unspeciated strain of *Bosia*, with 16% query coverage and 87% identity, suggesting potential homology for only a portion of the gene. No significant similarity was found to any of the other genes, suggesting that respiratory *Corynebacterium* strains may produce class IId bacteriocins that have not yet been characterized. Thus, while the *Corynebacterium* strains included in our study were not an abundant source of RiPPs, we identified gene clusters predicted to encode multiple bacteriocin classes, suggesting that novel RiPPs may be produced by respiratory *Corynebacterium* strains.

## DISCUSSION

In our investigation, mining whole-genome sequences from 148 non-diphtheriae respiratory *Corynebacterium* strains and 13 reference genomes uncovered 672 putative BGCs across multiple families. Additional BGC searches on genomes from other respiratory-associated bacterial species revealed a range in predicted biosynthetic capacity. *S. pneumoniae*, *S. mitis*, *H. influenzae*, *D. pigrum*, and *M. catarrhalis* harbored fewer BGC classes within their genomes, while a greater BGC class diversity was seen in the genomes of *C. diphtheriae*, *S. aureus*, and *S. epidermidis*, compared to the non-diphtheriae *Corynebacterium* genomes. The substantial number of BGCs identified in the relatively compact genomes of *Corynebacterium* species, in comparison to the majority of other common respiratory bacteria that we analyzed, suggests that secreted metabolites may serve as critical factors in the ecology of the human URT habitat. The diversity in biosynthetic potential among *Corynebacterium* species suggests that they may be a promising and underexplored source of secondary metabolites. Furthermore, this investigation substantially expands knowledge from previous studies on the potential biosynthetic capabilities of human-associated *Corynebacterium* species (53, 54).

The distribution of BGC classes appeared species-specific and varied at the strain level, as well as by the geographical source of the genome. This pattern may indicate a competitive intraspecies dynamic among *Corynebacterium* within the URT, particularly for species like *C. propinquum or C. pseudodiphtheriticum*, which showed more variation than *C. accolens*, *C. kefirresidentii*, or *C. marquesiae*. The stable and consistent representation of BGC profiles among frequent species like *C. accolens* may point to highly specific and adaptive mechanisms of niche assimilation among the diverse taxa in the microbiota. Species with little intraspecies variation may adapt to specific stable URT consortia, while *C. propinquum* or *C. pseudodiphtheriticum* may adapt to different consortia or URT microbial transitional states. BGCs were identified in every *Corynebacterium* genome analyzed in this study, suggesting that species of this genus possess rich biosynthetic capacities, regardless of the environments to which they are adapted. It is likely that secondary metabolites are utilized by *Corynebacterium* species in the URT as well as other environments.

Among the BGCs identified in non-diphtheriae *Corynebacterium* genomes, PKS and NRPS clusters were prevalent. These classes have historically been rich sources of natural antibiotic products and continue to be explored as sources of novel drugs. The abundance of PKS and NRPS clusters suggests that respiratory *Corynebacterium* strains likely produce a range of bioactive compounds, including polyketides and non-ribosomal peptides, which may confer ecological advantages by inhibiting competing bacteria. Predominantly T1PKS clusters were identified, with fewer putative type III and *trans*-AT clusters. Future research could compare a larger number of *Corynebacterium* isolates from different body sites to determine if the prevalence of PKS types correlates with specific niche adaptation.

Grouping analysis indicated that the coverage and amino acid identity between members of each T1PKS were high. However, for NRPS, while the coverage was high in each group, the amino acid identity varied greatly between groups, with groups 3 and 5 having close to 25% identity and groups 1, 2, and 4 having above 75% identity. The disparity in coverage vs amino acid identity can be explained by the fact that the two measurements are distinct. Group members with high coverage largely align over the length of the query gene, irrespective of sequence similarity. Conversely, the amino acid identity specifically reflects the degree of sequence similarity between the translated query gene and group members. Thus, while the sequences of the members may be similar to one another, they may share higher coverage than amino acid identity to the query. Overall, the grouping analyses demonstrated that there is a diversity of T1PKS and NRPS BGCs harbored by *Corynebacterium* species.

While less abundant than the PKS and NRPS BGCs, putative siderophore and RiPP clusters reside across respiratory strains belonging to multiple *Corynebacterium* species. The identification of siderophore BGCs in multiple species (including *C. pseudodiphtheriticum*, *C. kefirresidentii*, *C. marquesiae*, and unspeciated strains) expands on a previous study that found siderophore production in the species *C. propinquum* (17). Aside from their demonstrated importance in inter-bacterial competition, the importance of siderophores is further underscored by the growing interest in siderophore-conjugated antibiotics to combat antimicrobial resistance. These conjugates, such as the recently introduced cefiderocol, leverage bacterial iron uptake systems to introduce antibiotic molecules, potentially restoring the efficacy of drugs to which bacteria have developed resistance (55, 56). In addition to identifying NRPS-independent, IucA/IucC-like siderophores, other potential siderophore core biosynthetic genes included the gene *entF* encoding Enterobactin synthase component F, which was among the predicted NRPS core biosynthetic genes by antiSMASH6. This suggests that the capacity for siderophore production by *Corynebacterium* species may be greater than was previously known.

Recently, a *Corynebacterium lactis* strain was described that produces a novel bacteriocin with activity against other bacteria, primarily within the *Corynebacterium* genus (57). Our analysis identified eight distinct classes of RiPP BGCs among the *Corynebacterium* genomes, further supporting the possibility that other *Corynebacterium* species may also produce antibacterial RiPPs. Although no RiPPs are currently used as human therapeutics, the bacteriocin nisin has antimicrobial activity against bacteria responsible for food spoilage and has been widely utilized as a food preservative for decades (58, 59). The stability and strong antibacterial activities of RiPPs have sparked interest in their potential development as antibiotics for clinical use (60).

Limitations of this study lie primarily in the limits of *in silico* analysis. Tools like antiSMASH and GeneGrouper are invaluable for BGC identification and organization, but the clusters we identified are hypothetical until experimentally validated. The *in silico* analyses did not include prediction of mutations within cluster genes that could potentially alter or eliminate BGC function, nor did they comprehensively elucidate strain and species-level variation in gene sequences from shared clusters across members of a species. Additionally, identical or highly similar BGCs may have differential, conditional expression among species and strains, reflecting the energetically expensive nature of some metabolic products and their different contributions to niche maintenance and survival. Thus, while we have identified potential biosynthetic capabilities, the actual function and expression conditions of specific BGCs in the human URT remain to be elucidated. Finally, the number of strains per species varied, limiting the ability to exhaustively report the actual level of variation in BGC classes and the presence of BGCs within specific species.

This comprehensive exploration of BGCs among respiratory *Corynebacterium* strains reveals a compendium of factors that may alter human URT microbiota and pathobiont resistance through the production of inhibitory secondary metabolites. It also exposes a potentially rich source of novel antimicrobials within historically overlooked species of a significant bacterial genus. Furthermore, the inclusion of *Corynebacterium* genomes from non-respiratory sources and genomes from the pathogen *C. diphtheriae* in our analyses suggests that the potential to produce secondary metabolites is distributed among multiple *Corynebacterium* species and not restricted to commensal strains of the URT. This work should catalyze further functional studies on antimicrobial compounds produced by *Corynebacterium* strains. Our recent discovery that several nasopharyngeal *Corynebacterium* isolates secrete molecules inhibiting the globally important human pathobiont *S. pneumoniae* underscores the relevance of this study (7). The BGCs identified here could be instrumental in pinpointing these inhibitory substances. By advancing our understanding of the role of human-associated, commensal *Corynebacterium* species, this study sets the stage for the discovery of novel biotherapeutics derived from strains of this genus.

## MATERIALS AND METHODS

### Isolation and whole-genome sequencing of non-diphtheriae *Corynebacterium* strains from Duke University and Botswana

Sixty *Corynebacterium* strains were cultured from nasopharyngeal swab samples collected from mothers and infants in a birth cohort study conducted in Botswana, as previously described (7). Twelve *Corynebacterium* strains were cultured from patient samples of the upper or lower respiratory tract received by the Duke University Health System Clinical Microbiology Laboratory using the same methods. Genomic DNA was extracted from *Corynebacterium* strains using Powersoil Pro extraction kits (Qiagen) following the manufacturer’s instructions. DNA concentrations were determined using Qubit dsDNA high-sensitivity assay kits (Thermo Fisher Scientific). Library preparation was performed using Nextera XT DNA Library Preparation Kits (Illumina), and these libraries were sequenced on a NovaSeq 6000 instrument (Illumina) configured for 150 base pair paired-end reads. Adapter removal and read trimming were performed using Trimmomatic version 0.39 to a Phred score of 30 across a 4 bp sliding window; surviving reads shorter than 70 bp were discarded (61). The final quality of reads was assessed using FastQC version 0.11.9 (62). Genome assembly was performed using SPAdes version 3.15.3 (63).

### Isolation and whole-genome sequencing of non-diphtheriae *Corynebacterium* strains from Massachusetts

Nostril swabs were donated by adults and children participating in scientific outreach events in Massachusetts in 2017 and 2018 under an institutional review board-approved protocol, as previously described (40). Seventy-six *Corynebacterium* strains were isolated from swabs, and their genomes were sequenced as previously described (41).

### Genomic analyses of all non-diphtheriae *Corynebacterium* strains

The completeness of the genomes was evaluated with checkM version 1.1.3; all genomes were confirmed to have completeness above 95% and contamination below 5% (64). Genome sizes ranged from 2,155,492 bp to 2,977,329 bp, with an average size of 2,444,000 bp. For species assignment by ANI, the genomes of all strains were compared against publicly available genome sequences in the NCBI Genome database. The reference genome designated by NCBI was used for the comparison; all reference genomes corresponding to assigned *Corynebacterium* species used in this study had completeness above 95% and contamination below 5% and were downloaded from NCBI for subsequent BGC searches. However, not all reference genomes correspond to respiratory strains of *Corynebacterium* species. ANIs were calculated as previously described using FastANI (43). Strains with an ANI of ≥95% to a *Corynebacterium* reference genome were considered part of that species, following previously proposed standards (42, 65). Distinct strains were defined as having an ANI of less than 99.9% of all other genomes (66). All unspeciated strains had an ANI of >90% to a *Corynebacterium* reference genome, supporting their identification as being part of the genus based on an ~85% ANI species bound (43). The genome of the strain FDAARGOS_1055 was used as the reference to assign strains of *C. kefirresidentii*, which is not a validly published species. The non-diphtheriae respiratory *Corynebacterium* strains used in this study, along with the reference genomes downloaded from the NCBI Genome database, and their corresponding GenBank accession numbers are listed in Table S1.

antiSMASH6 was used to detect BGCs (44). All protocores from the BGCs identified by antiSMASH6 from non-diphtheriae respiratory *Corynebacterium* genomes and reference genomes are shown in Table S2. The uniqueness of the BGCs was determined by comparing the translated protein sequence from the predicted core gene of each BGC. GeneGrouper collapsed all the unique 66 T1PKS and (separately) 45 NRPS clusters into a smaller number of non-overlapping representative groups (45). Only unique, non-contig edge clusters were included in analyses performed using GeneGrouper. Only clusters on non-contig edges were used because if a BGC is found on a contig edge, the BGC is likely fragmented across multiple contigs, which can result in low-quality BGC data (67). All the unique genes identified from T1PKS or NRPS biosynthetic clusters were used as query (also called seed) sequences. Such genes were extracted from corresponding BGCs (T1PKS or NRPS), not from particular strains. Therefore, there were multiple seed genes used to take advantage of the whole core BGC content. GeneGrouper computes the Jaccard dissimilarity to assign group membership based on the gene content of the binned clusters. The Jaccard dissimilarity of each member relative to the group representative provides a measure of how different or dissimilar each member is compared to the representative sequence. A low Jaccard dissimilarity indicates high similarity between the member and the group representative. The full DNA sequence of each BGC analyzed and grouped by GeneGrouper was aligned and visualized by Easyfig (46). To create multiple sequence alignments, the translated core gene sequences of BGCs were aligned with the R package *msa* using the Muscle method (68, 69). The evolutionary history shown in the dendrograms was inferred using the neighbor-joining method (70). Phylogenetic analyses were conducted in MEGA X (71, 72).

### Selection and analysis of genomes from respiratory bacterial species

Genomes from the bacterial species *C. diphtheriae*, *D. pigrum*, *S. aureus*, *S. epidermidis*, *S. mitis*, *S. pneumoniae*, *M. catarrhalis*, and *H. influenzae* were obtained from NCBI and are listed in Table S3. The reference genome for each species was downloaded, in addition to up to 40 genomes from human respiratory (upper and lower respiratory tract) isolates. Only genomes with completeness >95% and contamination <5% were included. Each genome from a species was confirmed by ANI to be <99.9% similar to all other genomes and >95% similar to the reference genome, indicating that they came from distinct strains and were assigned to the correct species, respectively (43, 66). The genomes from respiratory strains, plus each reference genome, were mined by antiSMASH6. All protocore products of the BGCs identified by antiSMASH6 are shown in Table S4. For bacterial species that had more than 40 genomes from respiratory strains available, the genomes included in the analysis were randomly selected.

## Data Availability

The sequencing data sets supporting the conclusions of this study are available in the Sequence Read Archive (PRJNA804245, PRJNA842433, and PRJNA169440). The statistical files and script used for data analyses are publicly available at https://github.com/mskelly7/Coryne_genomics_manuscript.

## SUPPLEMENTAL MATERIAL

Figure S1 msphere.00258-25-s0001.tif Bar charts of *Corynebacterium* BGCs broken down by isolation source.

Figure S2 msphere.00258-25-s0002.pdf Gene homology of different T1PKS cluster groups.

Figure S3 msphere.00258-25-s0003.pdf Gene homology of different NRPS cluster groups.

Figure S4 msphere.00258-25-s0004.pdf Dendrogram/multiple sequence alignment of siderophore core biosynthesis enzymes.

Legends msphere.00258-25-s0005.docx Legends for Fig. S1-S4.

Table S1 msphere.00258-25-s0006.docx Non-diphtheriae *Corynebacterium* genomes used in this study.

Table S2 msphere.00258-25-s0007.xlsx Protcores of BGCs from non-diphtheriae *Corynebacterium* genomes used in this study.

Table S3 msphere.00258-25-s0008.docx Other respiratory bacterial species genomes used in this study.

Table S4 msphere.00258-25-s0009.xlsx BGC families and types identified from other respiratory bacterial genomes.

ASM does not own the copyrights to Supplemental Material that may be linked to, or accessed through, an article. The authors have granted ASM a non-exclusive, world-wide license to publish the Supplemental Material files. Please contact the corresponding author directly for reuse.
