# Supplementary figures and images for: Genome mining identifies a diversity of natural product biosynthetic capacity in human respiratory Corynebacterium strains
Source: mSphere. 2025 May 21;10(6):e00258-25. doi: 10.1128/msphere.00258-25 (PMC12188740; doi:10.1128/msphere.00258-25)

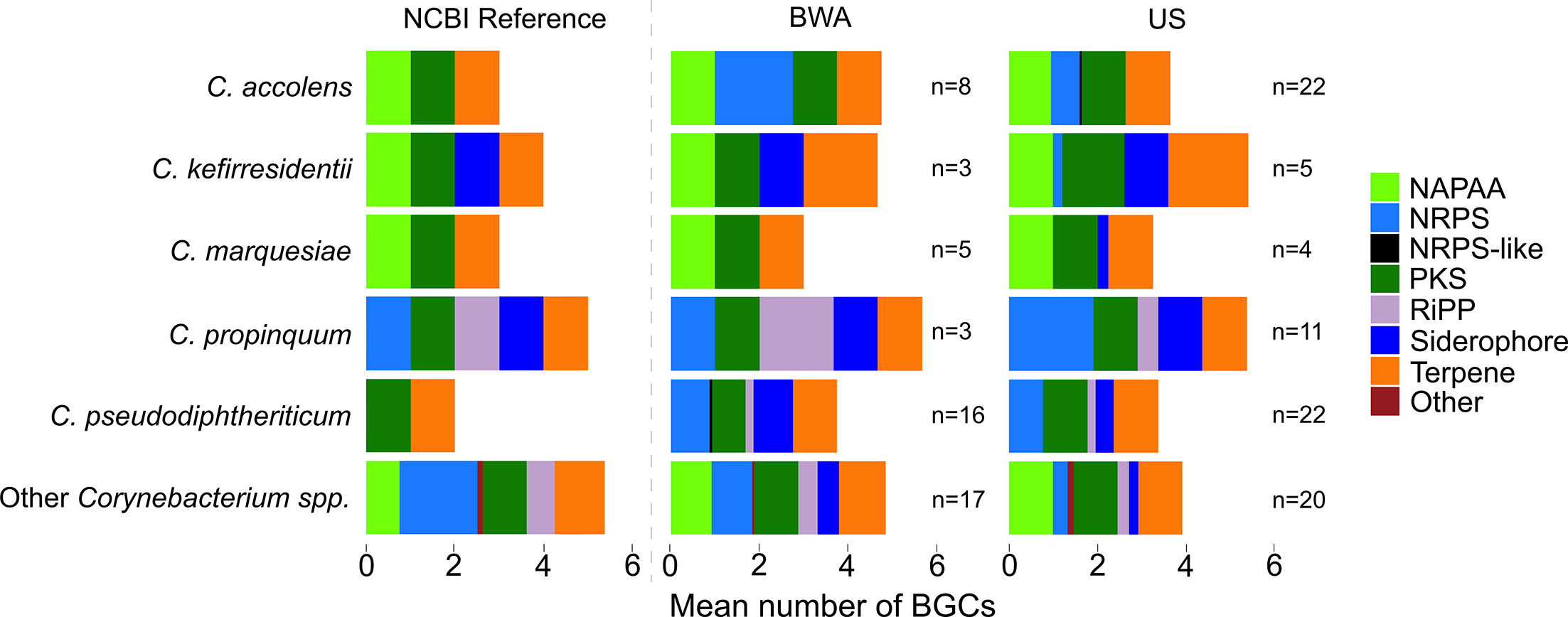

Supplement: Figure S1 — Bar charts of Corynebacterium BGCs broken down by isolation source. [file msphere.00258-25-s0001.tif]

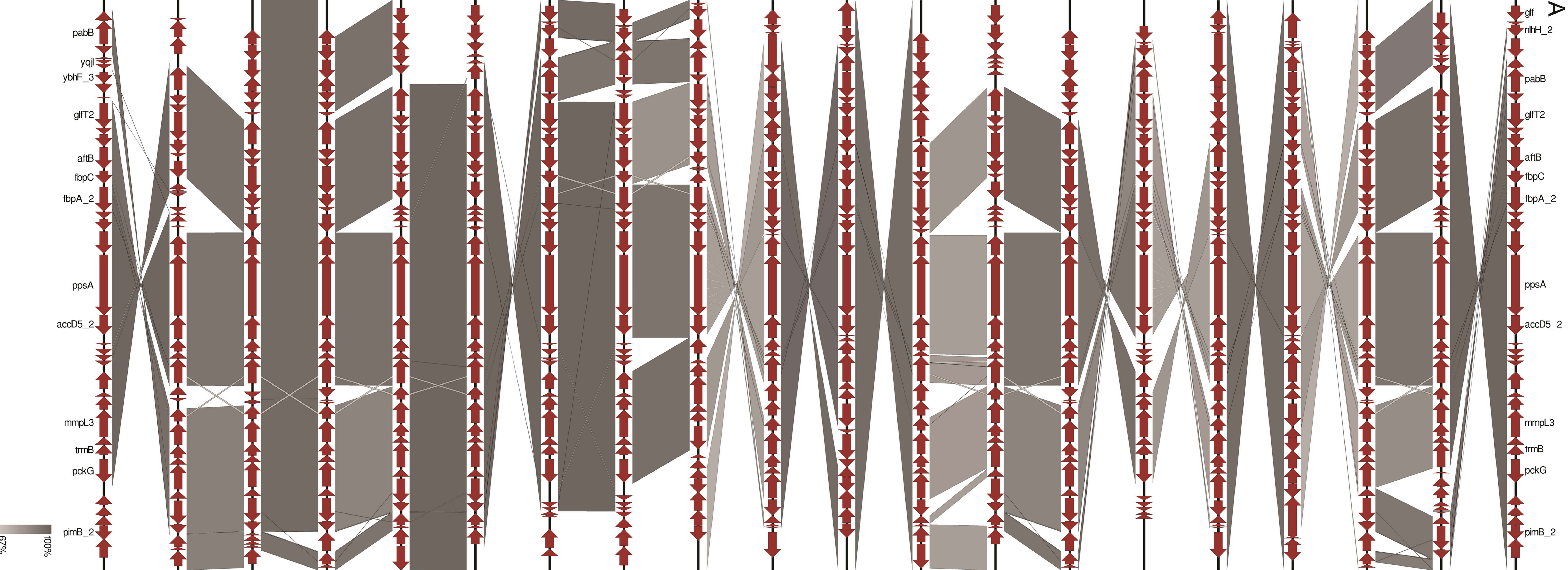

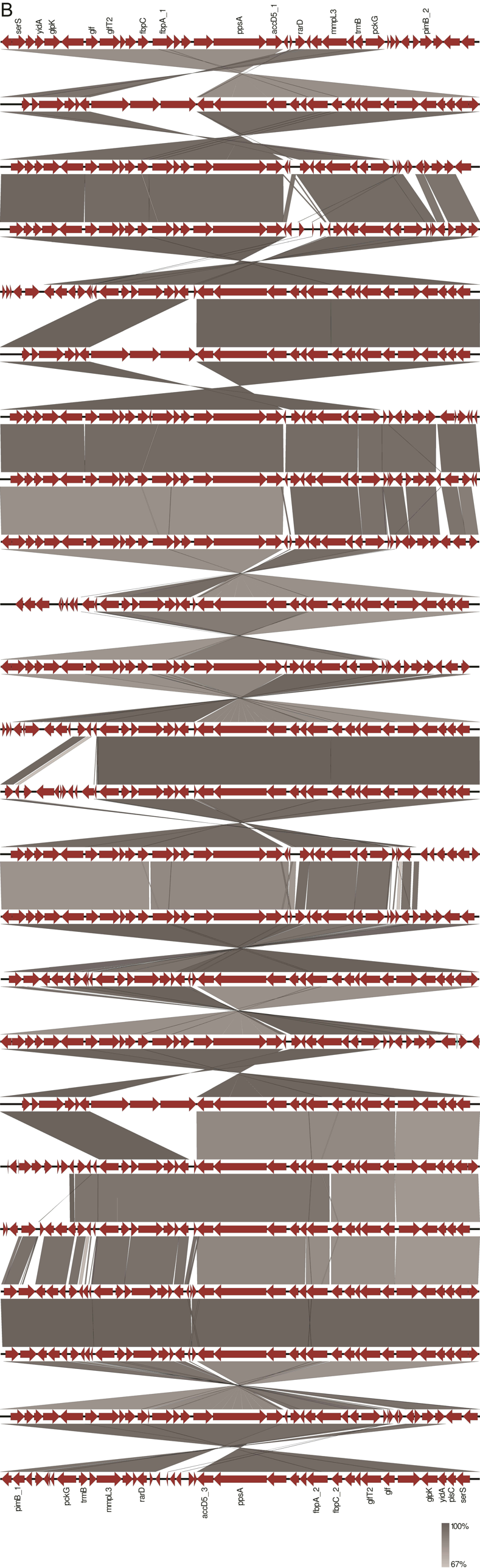

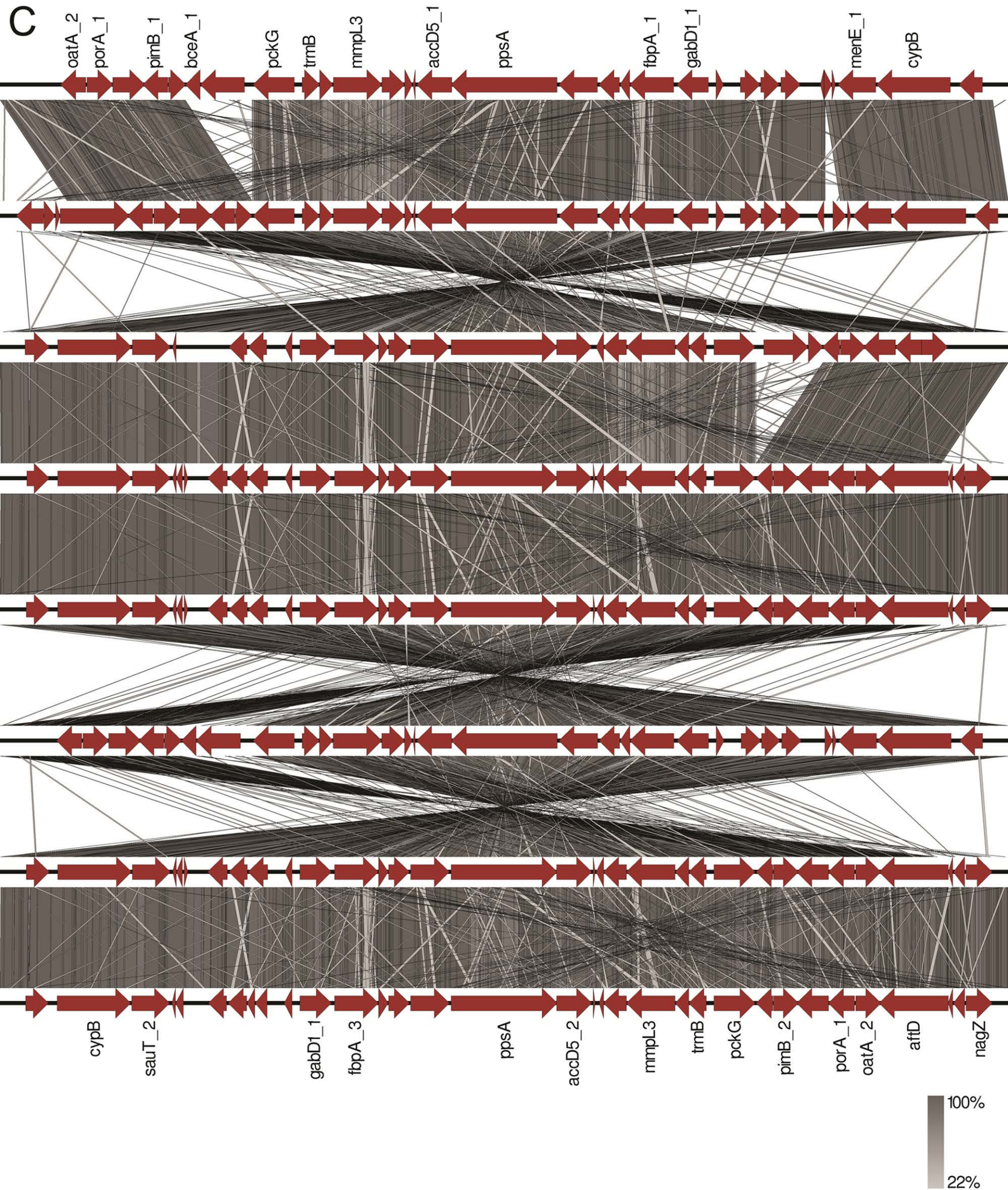

D

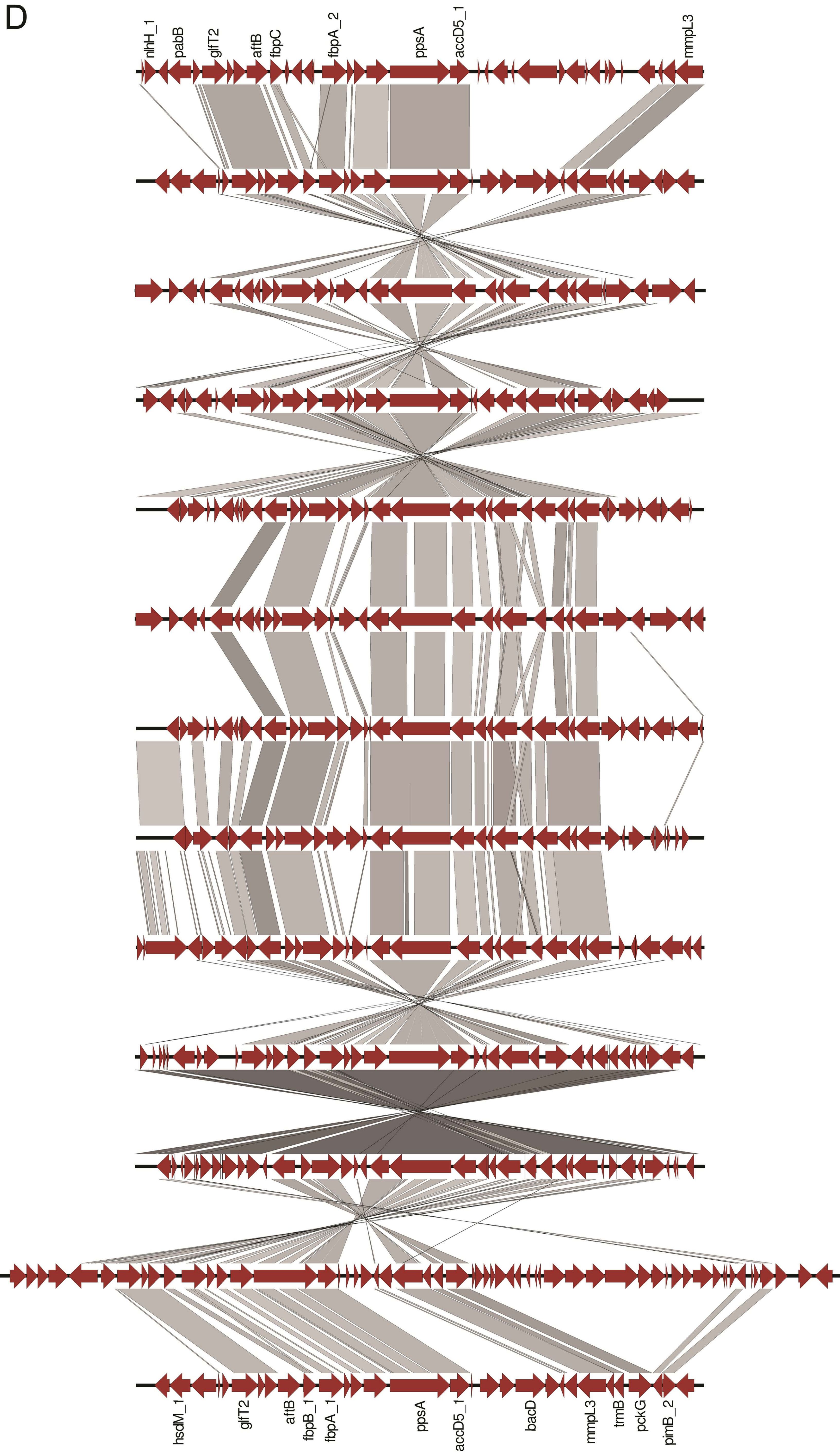

100%

65%

Supplement: Figure S2 — Gene homology of different T1PKS cluster groups. [file msphere.00258-25-s0002.pdf]

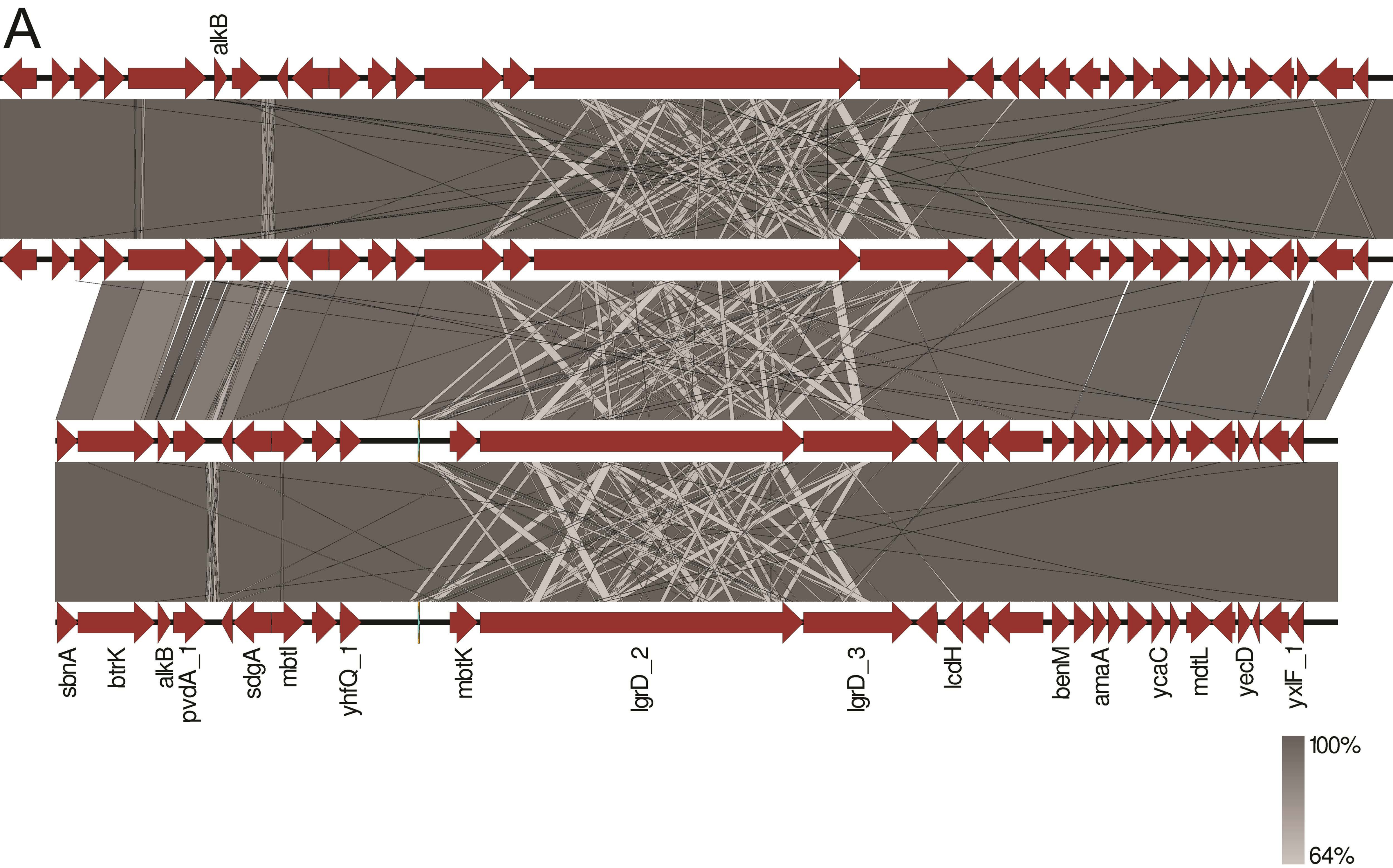

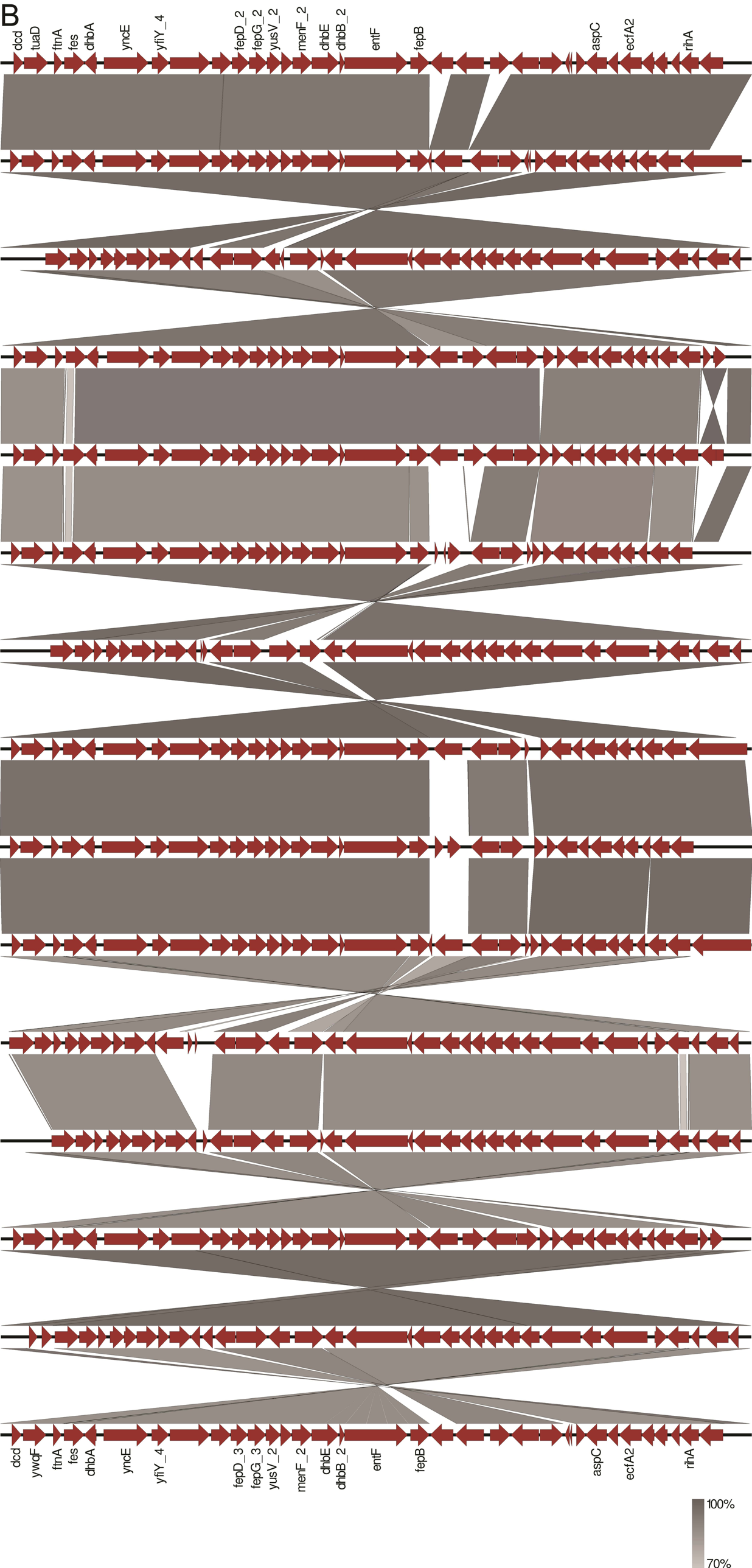

C

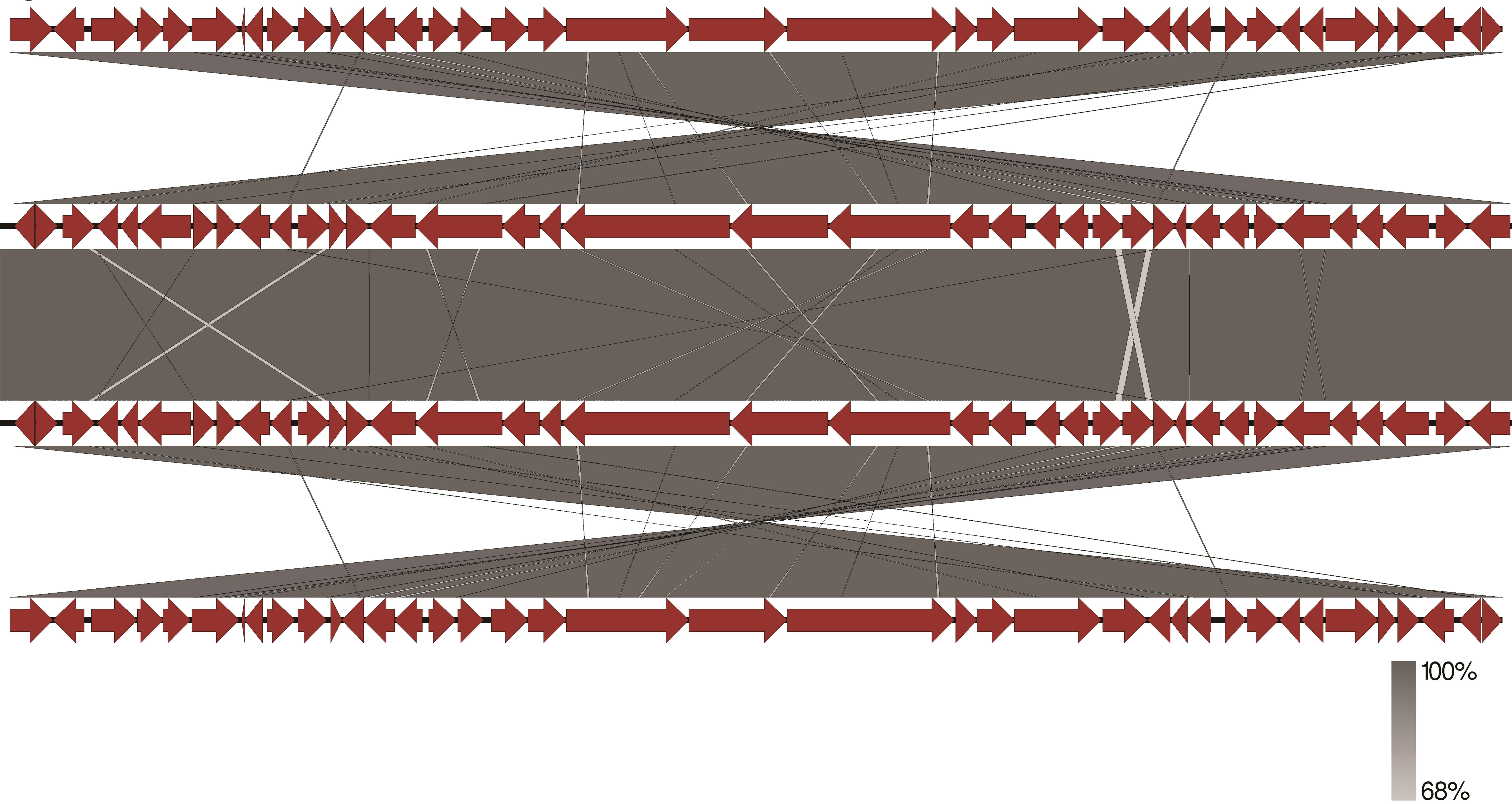

D

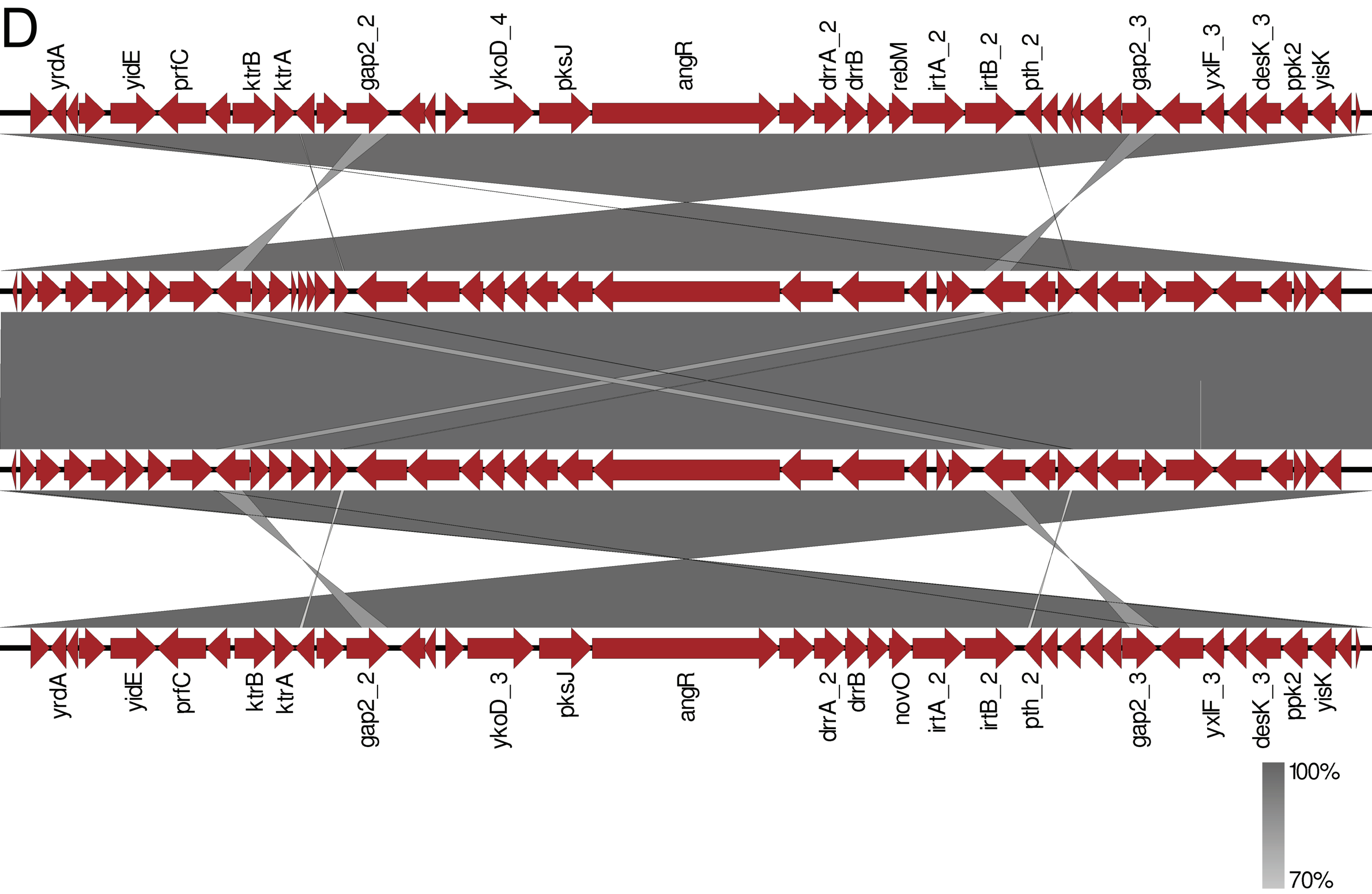

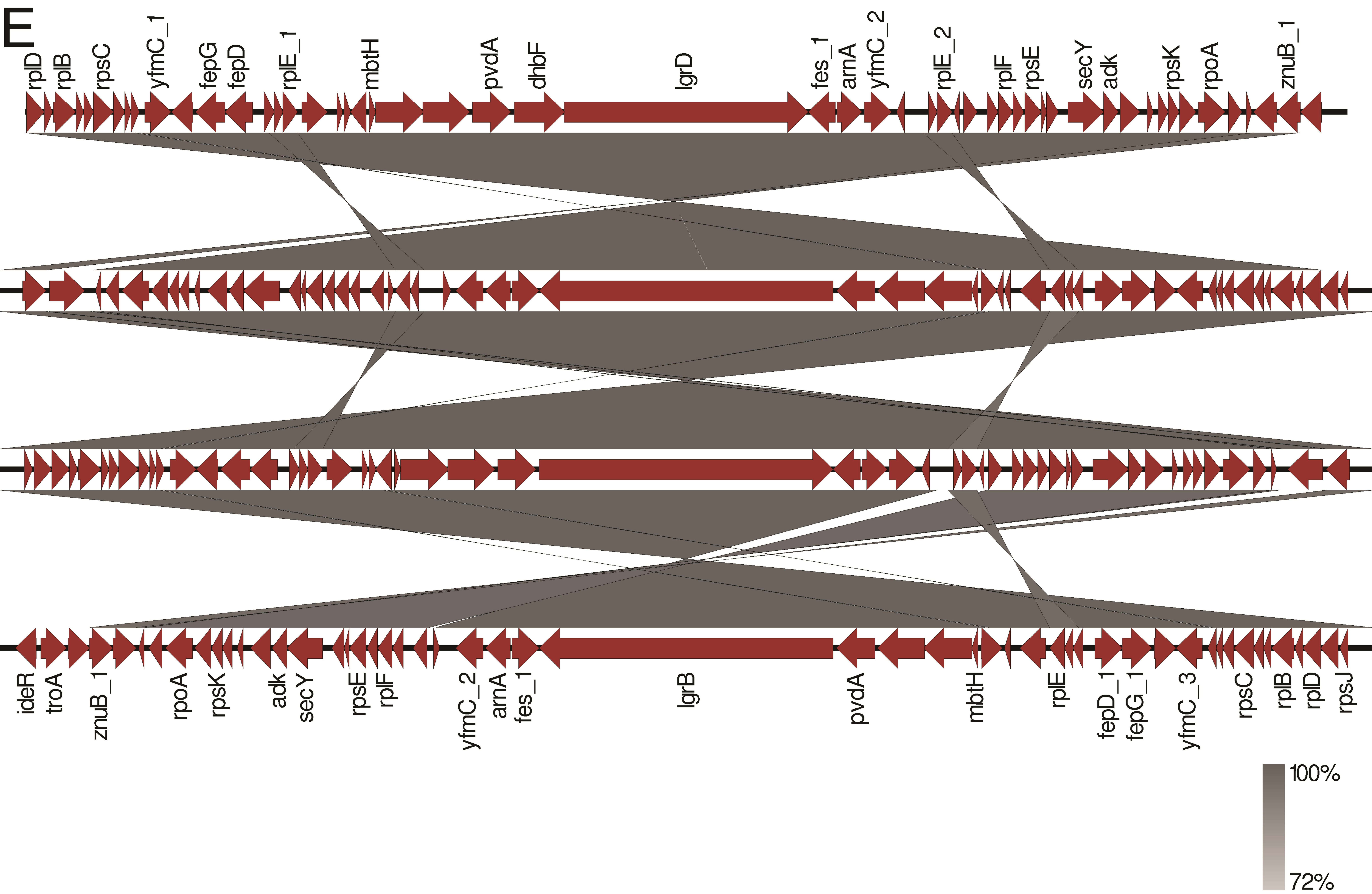

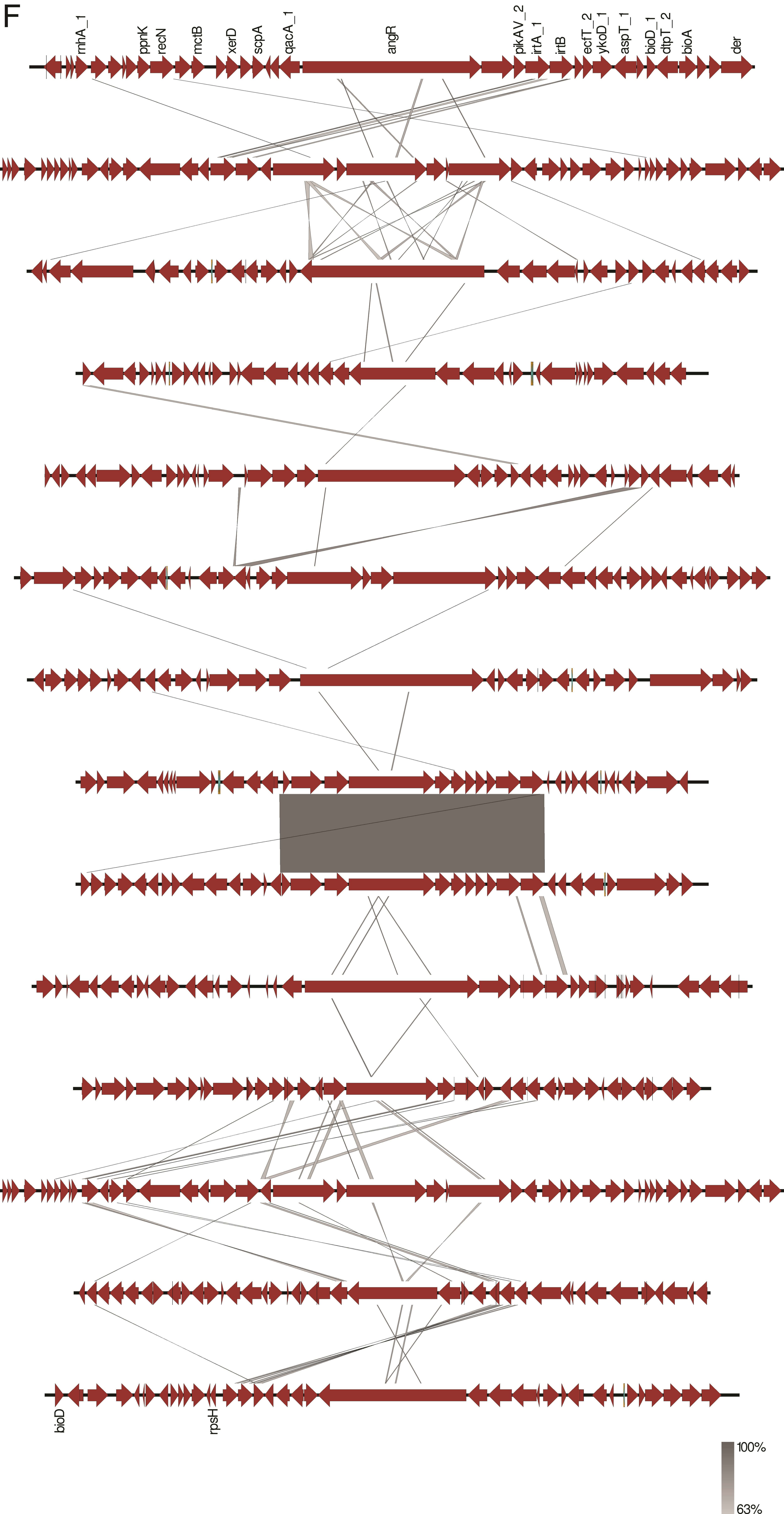

Supplement: Figure S3 — Gene homology of different NRPS cluster groups. [file msphere.00258-25-s0003.pdf]

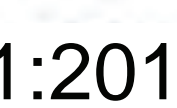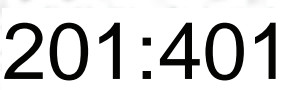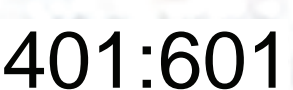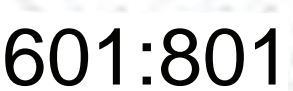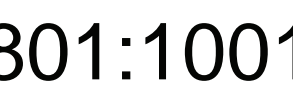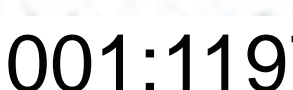

Supplement: Figure S4 — Dendrogram/multiple sequence alignment of siderophore core biosynthesis enzymes. [file msphere.00258-25-s0004.pdf]
