# Supplementary material for: Genome mining identifies a diversity of natural product biosynthetic capacity in human respiratory Corynebacterium strains
Source: mSphere. 2025 May 21;10(6):e00258-25. doi: 10.1128/msphere.00258-25 (PMC12188740; doi:10.1128/msphere.00258-25)
Supplement: Legends — for Fig. S1-S4. [file msphere.00258-25-s0005.docx]

**Supplemental Figure 1. Overview of the biosynthetic capacity in non-diphtheriae *Corynebacterium* strains broken down by isolation source.** (A) Bar plots depicting the mean number of BGCs for the most common BGC classes across the 13 reference genomes (NCBI reference), genomes from the respiratory strain collection isolated in Botswana (BWA), and genomes from the respiratory strain collection isolated in the United States (US). The number of genomes (written as n= values) representing each species in the BWA and US groups are included for reference. By definition, the NCBI reference genome is represented by an n of 1.

**Supplemental Figure 2. Visualization of the genetic similarity of T1PKS BGCs from non-diphtheriae *Corynebacterium* genomes categorized by GeneGrouper.** Alignment of the gene sequences of all T1PKS clusters categorized by GeneGrouper for each group. (A) Homology of all clusters from Group 1. (B) Homology of all clusters from Group 2. (C) Homology of all clusters from Group 3. (D) Homology of all clusters from the unbinned group. Putative gene annotations, where available, are included at the top and bottom of each panel. The degree of sequence homology between genes is demonstrated by the gray color saturation of the vertical blocks between each BGC and the range in homology is noted in the bottom right corner of each panel. The figures were generated with Easyfig.

**Supplemental Figure 3. Visualization of the genetic similarity of NRPS BGCs from non-diphtheriae *Corynebacterium* genomes categorized by GeneGrouper.** Alignment of the gene sequences of all NRPS clusters categorized by GeneGrouper for each group. (A) Homology of all clusters from Group 1. (B) Homology of all clusters from Group 2. (C) Homology of all clusters from Group 3. (D) Homology of all clusters from Group 4. (E) Homology of all clusters from Group 5. (F) Homology of all clusters from the unbinned group. Putative gene annotations, where available, are included at the top and bottom of each panel. The degree of sequence homology between genes is demonstrated by the gray color saturation of the vertical blocks between each BGC and the range in homology is noted in the bottom right corner of each panel. The figures were generated with Easyfig.

**Supplemental Figure 4. Multiple sequence alignment of the translated sequence of the core gene from siderophore BGCs.** Dendrogram and multiple sequence alignment of the amino acid translations of all unique predicted NIS synthetase core gene sequences identified by antiSMASH6. The evolutionary history was inferred using the neighbor-joining method. The percentage of replicate trees in which the associated taxa clustered together in the bootstrap test (500 replicates) are shown next to the branches (1). The evolutionary distances were computed using the Poisson correction method and are in the units of the number of amino acid substitutions per site (2).

**REFERENCES**

1. Felsenstein J. 1985. Confidence limits on phylogenies: an approach using the bootstrap. Evolution 39:783–791.

2. Zuckerkandl E, Pauling L. 1965. Evolutionary divergence and convergence in proteins, p. 97–166. *In* Bryson, V, Vogel, HJ (eds.), Evolving Genes and Proteins. Academic Press.
