# Supplementary material for: Genome mining identifies a diversity of natural product biosynthetic capacity in human respiratory Corynebacterium strains
Source: mSphere. 2025 May 21;10(6):e00258-25. doi: 10.1128/msphere.00258-25 (PMC12188740; doi:10.1128/msphere.00258-25)
Supplement: Table S1 — Non-diphtheriae Corynebacterium genomes used in this study. [file msphere.00258-25-s0006.docx]

**Supplemental Table 1. *Corynebacterium* strains used in this study***. Corynebacterium* strain identifications and their corresponding GenBank accession numbers, BioSample numbers, and final species assignments for all genomes used in this study. †Represents a reference genome.

| Strain | Assembly accession number | BioSample ID | Final Species | Isolation source |
| --- | --- | --- | --- | --- |
| KPL1818 | GCA_000478135.1 | SAMN02597007 | *C. accolens* | human respiratory tract |
| KPL1824 | GCA_000478095.1 | SAMN02597005 | *C. accolens* | human respiratory tract |
| KPL1855 | GCA_000478075.1 | SAMN02597004 | unclassified | human respiratory tract |
| KPL1989 | GCA_000477955.1 | SAMN02596998 | *C. pseudodiphtheriticum* | human respiratory tract |
| KPL1996 | GCA_000477915.1 | SAMN02596996 | *C. accolens* | human respiratory tract |
| KPL2617 | GCA_030176755.1 | SAMN35023380 | *C. accolens* | human respiratory tract |
| KPL2618 | GCA_030176885.1 | SAMN35023381 | *C. accolens* | human respiratory tract |
| KPL2619 | GCA_037908465.1 | SAMN39332962 | *C. yonathiae* | human respiratory tract |
| KPL2621 | GCA_030176735.1 | SAMN35023382 | *C. pseudodiphtheriticum* | human respiratory tract |
| KPL2636 | GCA_040224715.1 | SAMN41602962 | unclassified | human respiratory tract |
| KPL2640 | GCA_030176155.1 | SAMN35023383 | *C. pseudodiphtheriticum* | human respiratory tract |
| KPL2641 | GCA_030176875.1 | SAMN35023384 | *C. accolens* | human respiratory tract |
| KPL2652 | GCA_030176905.1 | SAMN35023385 | *C. accolens* | human respiratory tract |
| KPL2654 | GCA_030176855.1 | SAMN35023386 | *C. propinquum* | human respiratory tract |
| KPL2657 | GCA_030176765.1 | SAMN35023387 | *C. propinquum* | human respiratory tract |
| KPL2660 | GCA_030176015.1 | SAMN35023388 | *C. accolens* | human respiratory tract |
| KPL2667 | GCA_030176635.1 | SAMN35023389 | *C. pseudodiphtheriticum* | human respiratory tract |
| KPL2680 | GCA_040225675.1 | SAMN41602963 | unclassified | human respiratory tract |
| KPL2733 | GCA_030176715.1 | SAMN35023390 | *C. pseudodiphtheriticum* | human respiratory tract |
| KPL2734 | GCA_040225595.1 | SAMN41602965 | unclassified | human respiratory tract |
| KPL2755 | GCA_030176835.1 | SAMN35023391 | *C. propinquum* | human respiratory tract |
| KPL2773 | GCA_030176795.1 | SAMN35023392 | *C. pseudodiphtheriticum* | human respiratory tract |
| KPL2783 | GCA_030176785.1 | SAMN35023393 | *C. accolens* | human respiratory tract |
| KPL2795 | GCA_030176115.1 | SAMN35023395 | *C. pseudodiphtheriticum* | human respiratory tract |
| KPL2804 | GCA_030176195.1 | SAMN35023396 | *C. propinquum* | human respiratory tract |
| KPL2805 | GCA_040225415.1 | SAMN41602966 | unclassified | human respiratory tract |
| KPL2811 | GCA_030176025.1 | SAMN35023397 | *C. propinquum* | human respiratory tract |
| KPL2825 | GCA_037908445.1 | SAMN39332963 | *C. kefirresidentii* | human respiratory tract |
| KPL2826 | GCA_030176675.1 | SAMN35023398 | *C. pseudodiphtheriticum* | human respiratory tract |
| KPL2830 | GCA_040225635.1 | SAMN41602968 | unclassified | human respiratory tract |
| KPL2835 | GCA_040225575.1 | SAMN41602969 | *C. marquesiae* | human respiratory tract |
| KPL2838 | GCA_040225455.1 | SAMN41602970 | unclassified | human respiratory tract |
| KPL2850 | GCA_040225855.1 | SAMN41602972 | unclassified | human respiratory tract |
| KPL2859 | GCA_030176175.1 | SAMN35023400 | *C. accolens* | human respiratory tract |
| KPL2861 | GCA_040225475.1 | SAMN41602973 | unclassified | human respiratory tract |
| KPL2865 | GCA_030176135.1 | SAMN35023401 | *C. pseudodiphtheriticum* | human respiratory tract |
| KPL2866 | GCA_040225355.1 | SAMN41602974 | *C. marquesiae* | human respiratory tract |
| KPL2895 | GCA_040225815.1 | SAMN41602975 | unclassified | human respiratory tract |
| KPL2910 | GCA_040225435.1 | SAMN41602976 | *C. marquesiae* | human respiratory tract |
| KPL2915 | GCA_030176095.1 | SAMN35023402 | *C. pseudodiphtheriticum* | human respiratory tract |
| KPL3647 | GCA_030175965.1 | SAMN35023403 | *C. accolens* | human respiratory tract |
| KPL3649 | GCA_037908335.1 | SAMN39332964 | *C. kefirresidentii* | human respiratory tract |
| KPL3671 | GCA_030176615.1 | SAMN35023404 | *C. pseudodiphtheriticum* | human respiratory tract |
| KPL3672 | GCA_030176075.1 | SAMN35023405 | *C. propinquum* | human respiratory tract |
| KPL3674 | GCA_030175955.1 | SAMN35023406 | *C. accolens* | human respiratory tract |
| KPL3675 | GCA_030175975.1 | SAMN35023407 | *C. propinquum* | human respiratory tract |
| KPL3702 | GCA_030176535.1 | SAMN35023408 | *C. pseudodiphtheriticum* | human respiratory tract |
| KPL3703 | GCA_030176475.1 | SAMN35023409 | *C. accolens* | human respiratory tract |
| KPL3739 | GCA_040225515.1 | SAMN41602977 | unclassified | human respiratory tract |
| KPL3770 | GCA_030176595.1 | SAMN35023410 | *C. pseudodiphtheriticum* | human respiratory tract |
| KPL3772 | GCA_030176235.1 | SAMN35023411 | *C. pseudodiphtheriticum* | human respiratory tract |
| KPL3802 | GCA_030176515.1 | SAMN35023413 | *C. accolens* | human respiratory tract |
| KPL3804 | GCA_037908315.1 | SAMN39332965 | *C. kefirresidentii* | human respiratory tract |
| KPL3806 | GCA_040225935.1 | SAMN41602979 | unclassified | human respiratory tract |
| KPL3807 | GCA_030176495.1 | SAMN35023414 | unclassified | human respiratory tract |
| KPL3832 | GCA_030176575.1 | SAMN35023415 | *C. accolens* | human respiratory tract |
| KPL3833 | GCA_030176355.1 | SAMN35023416 | *C. pseudodiphtheriticum* | human respiratory tract |
| KPL3889 | GCA_030176435.1 | SAMN35023417 | *C. propinquum* | human respiratory tract |
| KPL3921 | GCA_030176375.1 | SAMN35023418 | *C. accolens* | human respiratory tract |
| KPL3926 | GCA_030176335.1 | SAMN35023419 | *C. accolens* | human respiratory tract |
| KPL3927 | GCA_040225835.1 | SAMN41602980 | unclassified | human respiratory tract |
| KPL3953 | GCA_030176415.1 | SAMN35023420 | *C. propinquum* | human respiratory tract |
| KPL3966 | GCA_030176395.1 | SAMN35023421 | *C. pseudodiphtheriticum* | human respiratory tract |
| KPL3967 | GCA_030176315.1 | SAMN35023422 | *C. propinquum* | human respiratory tract |
| KPL4010 | GCA_030176655.1 | SAMN35023424 | *C. pseudodiphtheriticum* | human respiratory tract |
| KPL4015 | GCA_040225725.1 | SAMN41602982 | unclassified | human respiratory tract |
| KPL4025 | GCA_030176295.1 | SAMN35023425 | *C. pseudodiphtheriticum* | human respiratory tract |
| KPL4034 | GCA_030176695.1 | SAMN35023426 | *C. accolens* | human respiratory tract |
| KPL4035 | GCA_037908325.1 | SAMN39332966 | *C. kefirresidentii* | human respiratory tract |
| KPL4040 | GCA_030175865.1 | SAMN35023427 | *C. propinquum* | human respiratory tract |
| KPL4041 | GCA_030176275.1 | SAMN35023428 | *C. pseudodiphtheriticum* | human respiratory tract |
| KPL4043 | GCA_040225715.1 | SAMN41602984 | unclassified | human respiratory tract |
| KPL4065 | GCA_030175915.1 | SAMN35023429 | *C. accolens* | human respiratory tract |
| KPL4066 | GCA_030175855.1 | SAMN35023430 | *C. pseudodiphtheriticum* | human respiratory tract |
| KPL4072 | GCA_037908395.1 | SAMN39332967 | *C. kefirresidentii* | human respiratory tract |
| KPL4083 | GCA_037908305.1 | SAMN39332968 | *C. marquesiae* | human respiratory tract |
| MSK037 | GCA_030232565.1 | SAMN35346693 | *C. pseudodiphtheriticum* | human respiratory tract |
| MSK050 | GCA_030232245.1 | SAMN35346699 | *C. striatum* | human respiratory tract |
| MSK064 | GCA_030232205.1 | SAMN35346702 | *C. striatum* | human respiratory tract |
| MSK065 | GCA_030232105.1 | SAMN35346703 | *C. striatum* | human respiratory tract |
| MSK067 | GCA_030232805.1 | SAMN35346704 | *C. striatum* | human respiratory tract |
| MSK070 | GCA_030232145.1 | SAMN35346705 | *C. accolens* | human respiratory tract |
| MSK071 | GCA_030232125.1 | SAMN35346706 | *C. kefirresidentii* | human respiratory tract |
| MSK072 | GCA_030232185.1 | SAMN35346707 | unclassified | human respiratory tract |
| MSK073 | GCA_030232085.1 | SAMN35346708 | unclassified | human respiratory tract |
| MSK074 | GCA_030229735.1 | SAMN35346709 | unclassified | human respiratory tract |
| MSK075 | GCA_030227275.1 | SAMN35346710 | *C. accolens* | human respiratory tract |
| MSK076 | GCA_030228895.1 | SAMN35346711 | *C. accolens* | human respiratory tract |
| MSK078 | GCA_030227155.1 | SAMN35346713 | unclassified | human respiratory tract |
| MSK079 | GCA_030228865.1 | SAMN35346714 | *C. accolens* | human respiratory tract |
| MSK080 | GCA_030227325.1 | SAMN35346715 | *C. pseudodiphtheriticum* | human respiratory tract |
| MSK081 | GCA_030227215.1 | SAMN35346716 | *C. mastitidis* | human respiratory tract |
| MSK082 | GCA_030229705.1 | SAMN35346717 | unclassified | human respiratory tract |
| MSK087 | GCA_030227265.1 | SAMN35346719 | *C. marquesiae* | human respiratory tract |
| MSK090 | GCA_030229725.1 | SAMN35346721 | unclassified | human respiratory tract |
| MSK092 | GCA_030229305.1 | SAMN35346723 | *C. pseudodiphtheriticum* | human respiratory tract |
| MSK095 | GCA_030229545.1 | SAMN35346725 | *C. appendicis* | human respiratory tract |
| MSK096 | GCA_030232755.1 | SAMN35346726 | *C. accolens* | human respiratory tract |
| MSK099 | GCA_030229625.1 | SAMN35346729 | *C. accolens* | human respiratory tract |
| MSK104 | GCA_030229005.1 | SAMN35346730 | *C. coyleae* | human respiratory tract |
| MSK109 | GCA_030229685.1 | SAMN35346733 | *C. accolens* | human respiratory tract |
| MSK110 | GCA_030229665.1 | SAMN35346734 | *C. pseudodiphtheriticum* | human respiratory tract |
| MSK118 | GCA_030232785.1 | SAMN35346735 | *C. pseudodiphtheriticum* | human respiratory tract |
| MSK121 | GCA_030229025.1 | SAMN35346736 | *C. accolens* | human respiratory tract |
| MSK122 | GCA_030229435.2 | SAMN35346737 | unclassified | human respiratory tract |
| MSK084 | GCA_030227345.1 | SAMN35346718 | unclassified | human respiratory tract |
| MSK124 | GCA_030228935.1 | SAMN35346739 | *C. propinquum* | human respiratory tract |
| MSK127 | GCA_030227205.1 | SAMN35346741 | *C. pseudodiphtheriticum* | human respiratory tract |
| MSK136 | GCA_022288805.2 | SAMN25718863 | *C. yonathiae* | human respiratory tract |
| MSK141 | GCA_030229485.1 | SAMN35346743 | *C. pseudodiphtheriticum* | human respiratory tract |
| MSK144 | GCA_030228985.1 | SAMN35346744 | *C. marquesiae* | human respiratory tract |
| MSK146 | GCA_030229105.1 | SAMN35346745 | *C. propinquum* | human respiratory tract |
| MSK150 | GCA_030229215.1 | SAMN35346746 | unclassified | human respiratory tract |
| MSK151 | GCA_030232645.1 | SAMN35346747 | unclassified | human respiratory tract |
| MSK156 | GCA_030232715.1 | SAMN35346748 | unclassified | human respiratory tract |
| MSK158 | GCA_030229085.1 | SAMN35346749 | unclassified | human respiratory tract |
| MSK161 | GCA_030229065.1 | SAMN35346750 | *C. accolens* | human respiratory tract |
| MSK163 | GCA_030229315.1 | SAMN35346751 | *C. kefirresidentii* | human respiratory tract |
| MSK164 | GCA_030232685.1 | SAMN35346752 | *C. pseudodiphtheriticum* | human respiratory tract |
| MSK166 | GCA_030232845.1 | SAMN35346753 | *C. pseudodiphtheriticum* | human respiratory tract |
| MSK168 | GCA_030229415.1 | SAMN35346754 | *C. pseudodiphtheriticum* | human respiratory tract |
| MSK172 | GCA_030229195.1 | SAMN35346755 | *C. pseudodiphtheriticum* | human respiratory tract |
| MSK173 | GCA_030232865.1 | SAMN35346756 | *C. freneyi* | human respiratory tract |
| MSK175 | GCA_030229125.1 | SAMN35346757 | unclassified | human respiratory tract |
| MSK180 | GCA_030229245.1 | SAMN35346758 | *C. bovis* | human respiratory tract |
| MSK184 | GCA_030229515.1 | SAMN35346759 | *C. pseudodiphtheriticum* | human respiratory tract |
| MSK185 | GCA_030229765.1 | SAMN35346760 | *C. kefirresidentii* | human respiratory tract |
| MSK188 | GCA_030229425.1 | SAMN35346761 | *C. pseudodiphtheriticum* | human respiratory tract |
| MSK189 | GCA_030229385.1 | SAMN35346762 | unclassified | human respiratory tract |
| MSK192 | GCA_030229905.1 | SAMN35346763 | unclassified | human respiratory tract |
| MSK195 | GCA_030228885.1 | SAMN35346765 | unclassified | human respiratory tract |
| MSK198 | GCA_030229835.1 | SAMN35346766 | *C. pseudodiphtheriticum* | human respiratory tract |
| MSK204 | GCA_030229885.1 | SAMN35346768 | unclassified | human respiratory tract |
| MSK207 | GCA_030229345.1 | SAMN35346769 | *C. tuberculostearicum* | human respiratory tract |
| MSK217 | GCA_030229865.1 | SAMN35346770 | *C. marquesiae* | human respiratory tract |
| MSK218 | GCA_030232655.1 | SAMN35346771 | unclassified | human respiratory tract |
| MSK223 | GCA_030229165.1 | SAMN35346773 | *C. pseudodiphtheriticum* | human respiratory tract |
| MSK270 | GCA_030229285.1 | SAMN35346775 | *C. accolens* | human respiratory tract |
| MSK273 | GCA_030229805.1 | SAMN35346776 | *C. accolens* | human respiratory tract |
| MSK281 | GCA_030228815.1 | SAMN35346777 | *C. marquesiae* | human respiratory tract |
| MSK283 | GCA_030228805.1 | SAMN35346778 | *C. pseudodiphtheriticum* | human respiratory tract |
| MSK290 | GCA_030229925.1 | SAMN35346779 | *C. propinquum* | human respiratory tract |
| MSK293 | GCA_030232745.1 | SAMN35346780 | unclassified | human respiratory tract |
| MSK297 | GCA_030232885.1 | SAMN35346781 | unclassified | human respiratory tract |
| MSK300 | GCA_030229145.1 | SAMN35346782 | *C. pseudodiphtheriticum* | human respiratory tract |
| MSK305 | GCA_030229035.1 | SAMN35346783 | *C. pseudodiphtheriticum* | human respiratory tract |
| MSK310 | GCA_030227355.1 | SAMN35346785 | unclassified | human respiratory tract |
| MSK315 | GCA_030227305.1 | SAMN35346788 | *C. marquesiae* | human respiratory tract |
| DSM 44278^†^ | GCA_023520795.1 | SAMN13404507 | *C. accolens* | human cervix |
| DSM 44287^†^ | GCA_000688415.1 | SAMN02743909 | *C. pseudodiphtheriticum* | human throat |
| c21Ua_68^†^ | GCA_027570195.1 | SAMN25350286 | *C. yonathiae* | human urine |
| FDAARGOS_1112^†^ | GCA_016728665.1 | SAMN16357281 | *C. propinquum* | human tracheal aspiration |
| FDAARGOS_1055^†^ | GCA_016599755.1 | SAMN16357224 | *C. kefirresidentii* | contaminant industrial laboratory sterility test |
| MSK093^†^ | GCA_030229635.1 | SAMN35346724 | *C. marquesiae* | human nasopharyngeal swab |
| FDAARGOS_1117^†^ | GCA_016728365.1 | SAMN16357286 | *C. tuberculostearicum* | human bone marrow |
| FDAARGOS_1115^†^ | GCA_016728105.1 | SAMN16357284 | *C. striatum* | eye |
| DSM 44356^†^ | GCA_000375365.1 | SAMN02441393 | *C. mastitidis* | milk of sheep with subclinical mastitis |
| CIP 107643^†^ | GCA_030408415.1 | SAMN03938400 | *C. appendicis* | human abdominal swab |
| FDAARGOS 1425^†^ | GCA_019048165.1 | SAMN16357567 | *C. coyleae* | human blood culture |
| FDAARGOS 1426^†^ | GCA_019047805.1 | SAMN16357568 | *C. freneyi* | pus of a human toe |
| 4826^†^ | GCA_003932295.1 | SAMN08139025 | *C. bovis* | milk of cow with mastitis |
