# Supplementary material for: Genome mining identifies a diversity of natural product biosynthetic capacity in human respiratory Corynebacterium strains
Source: mSphere. 2025 May 21;10(6):e00258-25. doi: 10.1128/msphere.00258-25 (PMC12188740; doi:10.1128/msphere.00258-25)
Supplement: Table S3 — Other respiratory bacterial species genomes used in this study. [file msphere.00258-25-s0008.docx]

**Supplemental Table 3. Other respiratory strains used in this study.** Species and strain identification and their corresponding GenBank accession numbers, BioSample numbers for all genomes used in this study. †Represents a reference genome.

| Strain | Assembly accession number | BioSample ID | Species | Isolation source |
| --- | --- | --- | --- | --- |
| 83VPs-KB5 | GCA_007197715.1 | SAMN12259797 | *Dolosigranulum pigrum* | nasal swab |
| KPL3050 | GCA_017655885.1 | SAMN11664192 | *D. pigrum* | nostril |
| KPL3090 | GCA_017655685.1 | SAMN11664200 | *D. pigrum* | nostril |
| KPL3052 | GCA_017655865.1 | SAMN11664193 | *D. pigrum* | nostril |
| KPL3069 | GCA_017655825.1 | SAMN11664195 | *D. pigrum* | nostril |
| KPL3033 | GCA_017655925.1 | SAMN11664190 | *D. pigrum* | nostril |
| KPL3246 | GCA_017655805.1 | SAMN11664201 | *D. pigrum* | nostril |
| KPL3084 | GCA_017655745.1 | SAMN11664198 | *D. pigrum* | nostril |
| KPL3250 | GCA_017655665.1 | SAMN11664202 | *D. pigrum* | nostril |
| KPL3911 | GCA_017655965.1 | SAMN11664206 | *D. pigrum* | nostril |
| KPL3043 | GCA_017655905.1 | SAMN11664191 | *D. pigrum* | nostril |
| KPL3070 | GCA_017655785.1 | SAMN11664196 | *D. pigrum* | nostril |
| KPL3065 | GCA_017655845.1 | SAMN11664194 | *D. pigrum* | nostril |
| KPL3274 | GCA_017655945.1 | SAMN11664205 | *D. pigrum* | nostril |
| AMBR12 | GCA_905071805.1 | SAMEA7487701 | *D. pigrum* | upper respiratory tract |
| KPL1922_CDC39-95 | GCA_003264145.1 | SAMN06624371 | *D. pigrum* | sinus |
| KPL1938_CDC4791-99 | GCA_003263975.1 | SAMN06624378 | *D. pigrum* | nasopharyngeal |
| KPL1939_CDC4792-99 | GCA_003263965.1 | SAMN06624379 | *D. pigrum* | nasopharyngeal |
| KPL1933_CDC4545-98 | GCA_003264045.1 | SAMN06624375 | *D. pigrum* | nasopharyngeal |
| KPL1930_CDC2949-98 | GCA_003264135.1 | SAMN06624372 | *D. pigrum* | nasopharyngeal |
| KPL3264 | GCA_017655705.1 | SAMN11664204 | *D. pigrum* | nostril |
| KPL3077 | GCA_017655765.1 | SAMN11664197 | *D. pigrum* | nostril |
| KPL3256 | GCA_017655645.1 | SAMN11664203 | *D. pigrum* | nostril |
| KPL1914 | GCA_003263915.2 | SAMN06621607 | *D. pigrum* | nose |
| ATCC 51524† | GCA_000245815.1 | SAMN02463904 | *D. pigrum* | spinal cord |
| R535 | GCA_001908955.1 | SAMN05938535 | *Haemophilus influenzae* | sputum |
| 5P28H1 | GCA_002966615.1 | SAMN06203638 | *H. influenzae* | sputum |
| 67P38H1 | GCA_002966655.1 | SAMN06203648 | *H. influenzae* | sputum |
| 6P24H2 | GCA_002966695.1 | SAMN06203655 | *H. influenzae* | sputum |
| 11P6H | GCA_002966735.1 | SAMN06203483 | *H. influenzae* | sputum |
| 93P28H1 | GCA_002988215.2 | SAMN06203705 | *H. influenzae* | sputum |
| P650-8603 | GCA_003425465.1 | SAMN07421927 | *H. influenzae* | sputum |
| P652-8881 | GCA_003425485.1 | SAMN07421929 | *H. influenzae* | sputum |
| P642-4396 | GCA_003425525.1 | SAMN07421919 | *H. influenzae* | sputum |
| P679-2791 | GCA_003425565.1 | SAMN07421955 | *H. influenzae* | sputum |
| P672-7661 | GCA_003425625.1 | SAMN07421947 | *H. influenzae* | sputum |
| P665-7858 | GCA_003425645.1 | SAMN07421940 | *H. influenzae* | sputum |
| P615-8618 | GCA_003425815.1 | SAMN07421892 | *H. influenzae* | sputum |
| P662-7189 | GCA_003425955.1 | SAMN07421937 | *H. influenzae* | sputum |
| 60316_BAL_Hi1 | GCA_004801985.1 | SAMN09845271 | *H. influenzae* | bronchoalveolar lavage |
| 60294_BAL_Hi1 | GCA_004802065.1 | SAMN09845267 | *H. influenzae* | bronchoalveolar lavage |
| 60295_BAL_Hi1 | GCA_004802095.1 | SAMN09845269 | *H. influenzae* | bronchoalveolar lavage |
| 60068_NP_Hi3 | GCA_004802145.1 | SAMN09845262 | *H. influenzae* | nasopharynx |
| 60051_NP_Hi1 | GCA_004802205.1 | SAMN09845260 | *H. influenzae* | nasopharynx |
| 60373_NP_Hi3 | GCA_004802285.1 | SAMN09845278 | *H. influenzae* | nasopharynx |
| 60362_NP_Hi1 | GCA_004802395.1 | SAMN09845272 | *H. influenzae* | nasopharynx |
| M1C112_1 | GCA_014931495.1 | SAMN16409221 | *H. influenzae* | cough swab |
| PT11370 | GCA_026158725.1 | SAMN27398392 | *H. influenzae* | nasopharynx |
| PT10978 | GCA_026159215.1 | SAMN27398375 | *H. influenzae* | nasopharynx |
| PT10993 | GCA_026159025.1 | SAMN27398379 | *H. influenzae* | nasopharynx |
| RMHi93 | GCA_000833735.1 | SAMN03284981 | *H. influenzae* | oropharynx |
| CCUG 26214 | GCA_001679235.1 | SAMN05214360 | *H. influenzae* | sputum |
| CCUG 54503 | GCA_001679365.1 | SAMN05219228 | *H. influenzae* | sputum |
| GE49 | GCA_002093185.1 | SAMN04590165 | *H. influenzae* | bronchoalveolar lavage |
| GE42 | GCA_002093195.1 | SAMN04590164 | *H. influenzae* | sputum |
| GE71 | GCA_002093255.1 | SAMN04590167 | *H. influenzae* | bronchoalveolar lavage |
| GE146 | GCA_002093275.1 | SAMN04590169 | *H. influenzae* | bronchial aspirate |
| GE117 | GCA_002093285.1 | SAMN04590168 | *H. influenzae* | bronchial aspirate |
| P588-8079 | GCA_003415495.2 | SAMN07421865 | *H. influenzae* | sputum |
| P617-9224 | GCA_003425715.1 | SAMN07421894 | *H. influenzae* | sputum |
| 65001_NP_Hi2 | GCA_004801995.1 | SAMN09845280 | *H. influenzae* | nasopharynx |
| 60373_NP_Hi1 | GCA_004802305.1 | SAMN09845276 | *H. influenzae* | nasopharynx |
| RHH-38 | GCA_010367335.1 | SAMN13942196 | *H. influenzae* | sputum |
| PT11407 | GCA_026158625.1 | SAMN27398396 | *H. influenzae* | nasopharynx |
| PT11604 | GCA_026155845.1 | SAMN27398402 | *H. influenzae* | nasopharynx |
| FDAARGOS_1560† | GCA_020736045.1 | SAMN22091657 | *H. influenzae* | unknown |
| CCUG 57283 | GCA_001679345.1 | SAMN05219449 | *Moraxella catarrhalis* | sputum |
| FDAARGOS_213 | GCA_002073215.2 | SAMN04875550 | *M. catarrhalis* | nasopharynx |
| FDAARGOS_304 | GCA_002984125.1 | SAMN06173317 | *M. catarrhalis* | nose |
| 142P87B1 | GCA_003971305.1 | SAMN10536190 | *M. catarrhalis* | sputum |
| 46P58B1 | GCA_003971325.1 | SAMN10536193 | *M. catarrhalis* | sputum |
| 74P58B1 | GCA_003971345.1 | SAMN10536192 | *M. catarrhalis* | sputum |
| 5P47B2 | GCA_003971365.1 | SAMN10536188 | *M. catarrhalis* | sputum |
| AS012774 | GCA_010603895.1 | SAMN12251093 | *M. catarrhalis* | lung |
| AS012772 | GCA_010603915.1 | SAMN12251091 | *M. catarrhalis* | lung |
| AS012768 | GCA_010603985.1 | SAMN12251087 | *M. catarrhalis* | lung |
| AS012766 | GCA_010604025.1 | SAMN12251085 | *M. catarrhalis* | lung |
| AS012765 | GCA_010604075.1 | SAMN12251084 | *M. catarrhalis* | lung |
| F18 | GCA_001656095.1 | SAMN04122790 | *M. catarrhalis* | sputum |
| F20 | GCA_001656105.1 | SAMN04122791 | *M. catarrhalis* | sputum |
| F23 | GCA_001656125.1 | SAMN04122793 | *M. catarrhalis* | sputum |
| F24 | GCA_001656195.1 | SAMN04122795 | *M. catarrhalis* | sputum |
| Z18 | GCA_001656255.1 | SAMN04122845 | *M. catarrhalis* | pharynx |
| C031 | GCA_001656385.1 | SAMN04122788 | *M. catarrhalis* | nose |
| N12 | GCA_001656455.1 | SAMN04122797 | *M. catarrhalis* | nose |
| N1 | GCA_001656475.1 | SAMN04122796 | *M. catarrhalis* | nose |
| N4 | GCA_001656505.1 | SAMN04122800 | *M. catarrhalis* | nose |
| CCUG 18283 | GCA_001679105.1 | SAMN05213945 | *M. catarrhalis* | nasopharynx |
| COPD_M127 | GCA_003625535.1 | SAMN09947558 | *M. catarrhalis* | sputum |
| COPD_M118 | GCA_003625545.1 | SAMN09947555 | *M. catarrhalis* | sputum |
| COPD_M70 | GCA_003625665.1 | SAMN09947539 | *M. catarrhalis* | sputum |
| T12 | GCA_009378105.1 | SAMN07672481 | *M. catarrhalis* | sputum |
| T4 | GCA_009378135.1 | SAMN07672482 | *M. catarrhalis* | sputum |
| S12N | GCA_022341255.1 | SAMN17178391 | *M. catarrhalis* | nasal mucosa |
| S15N | GCA_022341275.1 | SAMN17178392 | *M. catarrhalis* | nasal mucosa |
| S07N | GCA_022341325.1 | SAMN17178390 | *M. catarrhalis* | nasal mucosa |
| P17N | GCA_022341345.1 | SAMN17178388 | *M. catarrhalis* | nasal mucosa |
| P12N | GCA_022341385.1 | SAMN17178386 | *M. catarrhalis* | nasal mucosa |
| P15N | GCA_022341395.1 | SAMN17178387 | *M. catarrhalis* | nasal mucosa |
| C10 | GCA_001656415.1 | SAMN04122789 | *M. catarrhalis* | sputum |
| COPD_M121 | GCA_003625555.1 | SAMN09947556 | *M. catarrhalis* | sputum |
| COPD_M133 | GCA_003625595.1 | SAMN09947552 | *M. catarrhalis* | sputum |
| COPD_M16 | GCA_003625785.1 | SAMN09947519 | *M. catarrhalis* | sputum |
| COPD_M49 | GCA_003626335.1 | SAMN09947532 | *M. catarrhalis* | sputum |
| COPD_M37 | GCA_003626425.1 | SAMN09947527 | *M. catarrhalis* | sputum |
| COPD_M25 | GCA_003626455.1 | SAMN09947526 | *M. catarrhalis* | sputum |
| CCRI-195ME† | GCA_002080125.1 | SAMN05949222 | *M. catarrhalis* | middle ear |
| 17Gst354 | GCA_018135565.1 | SAMN12342460 | *Staphylococcus aureus* | nose |
| FORC_012 | GCA_001580495.1 | SAMN03387935 | *S. aureus* | sputum |
| N09CSA16 | GCA_024205325.1 | SAMN25263540 | *S. aureus* | sputum |
| TUM20818 | GCA_040931965.1 | SAMN42227009 | *S. aureus* | nasal swab |
| SA268 | GCA_000737615.1 | SAMN02261725 | *S. aureus* | sputum |
| C308 | GCA_027920385.1 | SAMN32538168 | *S. aureus* | sinus swab |
| TUM20819 | GCA_040931955.1 | SAMN42227010 | *S. aureus* | nasal swab |
| 2.3b | GCA_030643965.1 | SAMN34213606 | *S. aureus* | sputum |
| RIVM_M084526 | GCA_026547055.1 | SAMN27760541 | *S. aureus* | nose |
| S1-2-75 | GCA_024496845.1 | SAMN29924296 | *S. aureus* | sputum |
| BCH-SA-08 | GCA_003721005.1 | SAMN09847811 | *S. aureus* | throat culture |
| SA_5I | GCA_023621455.1 | SAMN28177556 | *S. aureus* | nasal carriage |
| CM16 | GCA_003240315.1 | SAMN08717617 | *S. aureus* | sputum |
| CM54 | GCA_003239625.1 | SAMN08717655 | *S. aureus* | sputum |
| CM112 | GCA_003239355.1 | SAMN08717713 | *S. aureus* | nasal swab |
| M17033 | GCA_000605265.1 | SAMN02402532 | *S. aureus* | nares |
| M121 | GCA_001021875.1 | SAMN02746692 | *S. aureus* | nasal swab |
| HC556 | GCA_001677355.1 | SAMN03737803 | *S. aureus* | nose secretion |
| C80 | GCA_001921695.1 | SAMN06077115 | *S. aureus* | nasal sample |
| USA300-SUR9 | GCA_002000565.1 | SAMN04497603 | *S. aureus* | nose |
| TW21 | GCA_002224925.1 | SAMN07340943 | *S. aureus* | nasal |
| BJ12 | GCA_015101775.1 | SAMN15887725 | *S. aureus* | pharynx swab |
| CM138 | GCA_003237215.1 | SAMN08717739 | *S. aureus* | bronchoaspiration material |
| CM135 | GCA_003237255.1 | SAMN08717736 | *S. aureus* | sputum |
| CM128 | GCA_003237335.1 | SAMN08717729 | *S. aureus* | nasal swab |
| CM127 | GCA_003237355.1 | SAMN08717728 | *S. aureus* | bronchoaspiration material |
| CM125 | GCA_003237365.1 | SAMN08717726 | *S. aureus* | bronchoaspiration material |
| CM109 | GCA_003237515.1 | SAMN08717710 | *S. aureus* | bronchoaspiration material |
| CM104 | GCA_003237535.1 | SAMN08717705 | *S. aureus* | bronchoaspiration material |
| CM103 | GCA_003237545.1 | SAMN08717704 | *S. aureus* | sputum |
| CM101 | GCA_003237595.1 | SAMN08717702 | *S. aureus* | bronchoaspiration material |
| CM93 | GCA_003237655.1 | SAMN08717694 | *S. aureus* | pharyngeal swab |
| CM88 | GCA_003237705.1 | SAMN08717689 | *S. aureus* | nasal swab |
| C0772 | GCA_003857315.1 | SAMN06920365 | *S. aureus* | oropharynx |
| C1512 | GCA_003857345.1 | SAMN06920367 | *S. aureus* | oropharynx |
| ZS_Z30 | GCA_003989645.1 | SAMN10638817 | *S. aureus* | nasal swab |
| NP7 | GCA_008040245.1 | SAMN12570107 | *S. aureus* | nasopharynx |
| NP6 | GCA_008040255.1 | SAMN12570106 | *S. aureus* | nasopharynx |
| NP2 | GCA_008040355.1 | SAMN12570101 | *S. aureus* | nasopharynx |
| NP17 | GCA_008040465.1 | SAMN12570100 | *S. aureus* | nasopharynx |
| NCTC 8325† | GCA_000013425.1 | SAMN02604235 | *S. aureus* | corneal ulcer |
| NCTC6513 | GCA_900458585.1 | SAMEA3512674 | *Staphylococcus epidermidis* | nose |
| 20925_1_31 | GCA_041432635.1 | SAMN30825743 | *S. epidermidis* | lung |
| 194 | GCA_035781615.1 | SAMN27533036 | *S. epidermidis* | oral swab |
| IVK83 | GCA_025809335.1 | SAMN23401985 | *S. epidermidis* | human nose |
| NIH051668 | GCA_000275985.2 | SAMN00993214 | *S. epidermidis* | lung |
| NCTC10519 | GCA_900458575.1 | SAMEA3871774 | *S. epidermidis* | nasal swab |
| p3-SID653 | GCA_025146855.1 | SAMN25653436 | *S. epidermidis* | nares swab |
| SE48 | GCA_003979905.1 | SAMN04526989 | *S. epidermidis* | nares |
| SEA37 | GCA_038116975.1 | SAMN36827138 | *S. epidermidis* | nasal cavity |
| CEMTC_2300 | GCA_020739975.1 | SAMN22599436 | *S. epidermidis* | nose |
| X6293 | GCA_038502265.1 | SAMN37641183 | *S. epidermidis* | nasal |
| HESN035b | GCA_015070555.1 | SAMN16402353 | *S. epidermidis* | nasal mucosa |
| 14.1.R1 | GCA_022815765.1 | SAMN27064491 | *S. epidermidis* | nasal swab |
| CCSH-141 | GCA_030435975.1 | SAMN36269256 | *S. epidermidis* | nasal cavity |
| SNS5pa | GCA_035780455.1 | SAMN27533073 | *S. epidermidis* | nasal swab |
| INNS29 | GCA_035780555.1 | SAMN27533069 | *S. epidermidis* | nasal swab |
| NC77 | GCA_035780755.1 | SAMN27533066 | *S. epidermidis* | nasal swab |
| NNS11w | GCA_035785895.1 | SAMN27533560 | *S. epidermidis* | nasal swab |
| NNS5pa | GCA_035785955.1 | SAMN27533557 | *S. epidermidis* | nasal swab |
| NNS8w | GCA_035785975.1 | SAMN27533555 | *S. epidermidis* | nasal swab |
| NC71 | GCA_035787255.1 | SAMN27533489 | *S. epidermidis* | nasal swab |
| 4501 | GCA_035790335.1 | SAMN28910824 | *S. epidermidis* | nasal swab |
| 4471 | GCA_035790395.1 | SAMN28910821 | *S. epidermidis* | nasal swab |
| X5485 | GCA_038501825.1 | SAMN37641177 | *S. epidermidis* | nasal |
| X6049b | GCA_043848455.1 | SAMN37641182 | *S. epidermidis* | nasal |
| X6590 | GCA_043848475.1 | SAMN37641178 | *S. epidermidis* | nasal |
| X9066 | GCA_043848495.1 | SAMN37641180 | *S. epidermidis* | nasal |
| 20925_1_59 | GCA_041432645.1 | SAMN30825744 | *S. epidermidis* | lung |
| C10C | GCA_000763625.1 | SAMN02996614 | *S. epidermidis* | sputum |
| SAM-3 | GCA_003038655.1 | SAMN08683515 | *S. epidermidis* | sputum |
| FFMG-199-8-21 | GCA_964210225.1 | SAMEA115875195 | *S. epidermidis* | sputum |
| SE53 | GCA_003979985.1 | SAMN04526994 | *S. epidermidis* | nares |
| SE52 | GCA_003979975.1 | SAMN04526993 | *S. epidermidis* | nares |
| X6628 | GCA_038377765.1 | SAMN37641179 | *S. epidermidis* | nasal |
| B273 | GCA_041080605.1 | SAMN42914137 | *S. epidermidis* | anterior nares |
| NIHLM061 | GCA_000276445.1 | SAMN00993260 | *S. epidermidis* | nare |
| NIHLM087 | GCA_000276505.1 | SAMN00991849 | *S. epidermidis* | nare |
| ATCC 14990† | GCA_006094375.1 | SAMN10738425 | *S. epidermidis* | nose |
| OT25 | GCA_000960005.1 | SAMN03334899 | *Streptococcus mitis* | oral cavity |
| BCC65 | GCA_003942775.1 | SAMN09631740 | *S. mitis* | dental plaque |
| BCC44 | GCA_003942885.1 | SAMN09631733 | *S. mitis* | dental plaque |
| BCC06 | GCA_003942995.1 | SAMN09631728 | *S. mitis* | dental plaque |
| SM41 | GCA_009496235.1 | SAMN13105981 | *S. mitis* | oral swab |
| 103U_S92 | GCA_946186075.1 | SAMEA14489604 | *S. mitis* | respiratory tract |
| U104_S93 | GCA_946188685.1 | SAMEA14489561 | *S. mitis* | respiratory tract |
| WTCHG_774611_73075379 | GCA_946190295.1 | SAMEA110470963 | *S. mitis* | nasopharynx |
| KCOM 1350 (= ChDC B183)† | GCA_001281025.1 | SAMN03263069 | *S. mitis* | oral cavity |
| PT8105 | GCA_001095405.3 | SAMEA1463109 | *Streptococcus pneumoniae* | nasopharynx |
| 6A-10 | GCA_013047165.1 | SAMN14779717 | *S. pneumoniae* | nasopharynx |
| BVJ1JL | GCA_017569245.1 | SAMN17602804 | *S. pneumoniae* | nasopharynx |
| SCAID PHRX1-2021 | GCA_019915285.1 | SAMN20982453 | *S. pneumoniae* | pharynx swab |
| TVO_1902282 | GCA_022070565.1 | SAMN10734538 | *S. pneumoniae* | nasopharynx |
| BR1268 | GCA_022318385.1 | SAMN08647280 | *S. pneumoniae* | nasopharyngeal |
| CH2241 | GCA_022318405.1 | SAMN08647361 | *S. pneumoniae* | nasopharyngeal |
| NP7513 | GCA_022318485.1 | SAMN08647831 | *S. pneumoniae* | nasopharyngeal |
| PT8465 | GCA_022318525.1 | SAMN08647902 | *S. pneumoniae* | nasopharyngeal |
| 19A-ST320_99-176 | GCA_025136435.1 | SAMN16580452 | *S. pneumoniae* | sputum |
| 16H2092 | GCA_033144075.1 | SAMN37865677 | *S. pneumoniae* | throat swab |
| 16P4028 | GCA_033144835.1 | SAMN37865676 | *S. pneumoniae* | throat swab |
| 15H4024 | GCA_033146375.1 | SAMN37865675 | *S. pneumoniae* | throat swab |
| 16H3071-2 | GCA_033147055.1 | SAMN37865680 | *S. pneumoniae* | throat swab |
| 16P2012-2 | GCA_033147875.1 | SAMN37865681 | *S. pneumoniae* | throat swab |
| 20824-4 | GCA_033148655.1 | SAMN37865683 | *S. pneumoniae* | throat swab |
| 02H2025 | GCA_033149375.1 | SAMN37865670 | *S. pneumoniae* | throat swab |
| 16H2041 | GCA_033150235.1 | SAMN37865672 | *S. pneumoniae* | throat swab |
| 17023 | GCA_033151055.1 | SAMN37865671 | *S. pneumoniae* | sputum |
| 15P3054 | GCA_033152035.1 | SAMN37865668 | *S. pneumoniae* | throat swab |
| 20614-6 | GCA_033152795.1 | SAMN37865667 | *S. pneumoniae* | throat swab |
| 11012 | GCA_033167795.1 | SAMN37865673 | *S. pneumoniae* | throat swab |
| 21011 | GCA_034422155.1 | SAMN38608486 | *S. pneumoniae* | sputum |
| 16H2017-2 | GCA_034422215.1 | SAMN38608497 | *S. pneumoniae* | throat swab |
| 05H0020-2 | GCA_034422275.1 | SAMN38608520 | *S. pneumoniae* | throat swab |
| 15P2112 | GCA_034422335.1 | SAMN38608524 | *S. pneumoniae* | throat swab |
| 2018N21-288 | GCA_038431325.1 | SAMN36766248 | *S. pneumoniae* | respiratory |
| 2008C09-280 | GCA_038431755.1 | SAMN36766242 | *S. pneumoniae* | respiratory |
| 2008C09-276 | GCA_038431765.1 | SAMN36766241 | *S. pneumoniae* | respiratory |
| 16P29 | GCA_041154405.1 | SAMD00622274 | *S. pneumoniae* | sputum |
| 6_2F1 | GCA_041955655.1 | SAMN43413908 | *S. pneumoniae* | oropharynx |
| NCTC12977 | GCA_900636585.1 | SAMEA3679170 | *S. pneumoniae* | sputum |
| SMRU965 | GCA_001170545.1 | SAMEA1024876 | *S. pneumoniae* | nasopharynx |
| ASVL_JC_0001 | GCA_000685585.1 | SAMN02716923 | *S. pneumoniae* | throat |
| SMRU560 | GCA_000832325.1 | SAMEA1023802 | *S. pneumoniae* | nasopharynx |
| SMRU557 | GCA_000939355.1 | SAMEA1023754 | *S. pneumoniae* | nasopharynx |
| SMRU563 | GCA_000941155.1 | SAMEA1023830 | *S. pneumoniae* | nasopharynx |
| SMRU554 | GCA_000942775.1 | SAMEA1023675 | *S. pneumoniae* | nasopharynx |
| SMRU562 | GCA_000944255.1 | SAMEA1023843 | *S. pneumoniae* | nasopharynx |
| SMRU556 | GCA_000945715.1 | SAMEA1023703 | *S. pneumoniae* | nasopharynx |
| NCTC7465† | GCA_001457635.1 | SAMEA2479568 | *S. pneumoniae* | unknown |
| ST378-KZN-2015-45791 | GCA_001875805.1 | SAMN05729416 | *Corynebacterium diphtheriae* | throat and nasal swab |
| ST395-KZN-2015-45792 | GCA_001875875.1 | SAMN05729417 | *C. diphtheriae* | throat and nasal swab |
| B-D-16-78 | GCA_001902275.1 | SAMN06099113 | *C. diphtheriae* | nasopharyngeal swab |
| BQ11 | GCA_003194045.1 | SAMN09269643 | *C. diphtheriae* | pharyngeal swab |
| TH1526 | GCA_004758745.1 | SAMN05615426 | *C. diphtheriae* | throat swab |
| CD1009 | GCA_021560115.1 | SAMN11612662 | *C. diphtheriae* | throat swab |
| CD1075 | GCA_021560215.1 | SAMN11612667 | *C. diphtheriae* | nasal swab |
| CD1040 | GCA_021560235.1 | SAMN11612666 | *C. diphtheriae* | throat swab |
| CD1036 | GCA_021560255.1 | SAMN11612665 | *C. diphtheriae* | throat swab |
| CD1032 | GCA_021560295.1 | SAMN11612663 | *C. diphtheriae* | throat swab |
| NML181114 | GCA_023652155.1 | SAMN25124811 | *C. diphtheriae* | throat |
| GMR-D-100.18 | GCA_017149575.1 | SAMN15693636 | *C. diphtheriae* | pharyngeal swab |
| WD62 | GCA_003666645.1 | SAMN10186723 | *C. diphtheriae* | throat swab |
| c110 | GCA_001832975.1 | SAMN05877644 | *C. diphtheriae* | nasal discharge |
| rz632 | GCA_002115145.1 | SAMN06311218 | *C. diphtheriae* | throat swab |
| rz597 | GCA_002115185.1 | SAMN06311216 | *C. diphtheriae* | throat swab |
| 03-15 | GCA_015830215.1 | SAMN14209808 | *C. diphtheriae* | throat |
| LACPHL-SPEC-2024-00436 | GCA_041450195.1 | SAMN43227682 | *C. diphtheriae* | throat |
| 11-14 | GCA_015830305.1 | SAMN14209804 | *C. diphtheriae* | sputum |
| rz358 | GCA_001889855.1 | SAMN05877638 | *C. diphtheriae* | throat swab |
| NC03529 | GCA_000257885.1 | SAMN02471955 | *C. diphtheriae* | throat |
| NCTC 5011 | GCA_000263415.1 | SAMN02471953 | *C. diphtheriae* | throat swab |
| 17801 | GCA_000968865.1 | SAMN03396925 | *C. diphtheriae* | throat swab |
| c123 | GCA_001832925.1 | SAMN05877646 | *C. diphtheriae* | throat swab |
| c325 | GCA_001832935.1 | SAMN05877647 | *C. diphtheriae* | throat swab |
| 28-14 | GCA_015830285.1 | SAMN14209805 | *C. diphtheriae* | wound |
| 18-13 | GCA_015830385.1 | SAMN14209799 | *C. diphtheriae* | nasal |
| 632/19 | GCA_024969545.1 | SAMN30508160 | *C. diphtheriae* | respiratory tracks |
| rz356 | GCA_001889775.1 | SAMN05877637 | *C. diphtheriae* | throat swab |
| rz373 | GCA_001889825.1 | SAMN05877639 | *C. diphtheriae* | throat swab |
| rz693 | GCA_002115095.1 | SAMN06311222 | *C. diphtheriae* | throat swab |
| rz656 | GCA_002115125.1 | SAMN06311219 | *C. diphtheriae* | throat swab |
| NCTC11397† | GCA_001457455.1 | SAMEA2517360 | *C. diphtheriae* | unknown |
|  |  |  |  |  |
|  |  |  |  |  |
|  |  |  |  |  |
|  |  |  |  |  |
|  |  |  |  |  |
|  |  |  |  |  |
